# Efficacy and safety of 5-aminolevulinic acid photodynamic therapy for cervical squamous intraepithelial lesion: a systematic review and meta-analysis

**DOI:** 10.1007/s10103-026-04955-9

**Published:** 2026-07-18

**Authors:** Weilin Guo, Ruiju He, Yuan Hu, Kaiyan Gu, Lihua Qiu

**Affiliations:** 1 Department of Obstetrics and Gynecology, Ren Ji Hospital, Shanghai Jiao Tong University School of Medicine, Shanghai, China; 2 Shanghai Key Laboratory of Gynecologic Oncology, Ren Ji Hospital, Shanghai Jiao Tong University School of Medicine, Shanghai, China

**Keywords:** 5-aminolevulinic acid, Photodynamic therapy, Cervical squamous intraepithelial lesion, Human papillomavirus

## Abstract

To evaluate the efficacy and safety of 5-aminolevulinic acid photodynamic therapy (5-ALA PDT) for cervical squamous intraepithelial lesion (SIL). We systematically searched PubMed and other databases to compare the effectiveness of 5-ALA PDT with observation, ablation, and conization. Outcomes included overall response rate (ORR), complete remission (CR) rate, HPV clearance rate, and other relevant endpoints. Single-arm analyses were additionally performed by lesion grade. A total of 3,354 patients were included across 19 studies. The ORR with PDT was significantly higher than with observation (OR = 10.27, 95% CI: 3.87–27.27) and ablation (OR = 2.57, 95% CI: 2.00-3.30), with no significant difference compared with conization. At 6 months, HPV clearance rate was significantly higher with PDT than with observation (OR = 11.47, 95% CI: 3.16–41.68) and ablation (OR = 1.46, 95% CI: 1.01–2.13), but was comparable to conization. PDT also associated with a lower risk of progression than observation (OR = 0.19, 95% CI: 0.11–0.34) and a lower recurrence rate than ablation (OR = 0.30, 95%CI 0.14–0.63). ORR was 0.86 (95% CI: 0.83–0.89) in LSIL and 0.91 (95% CI: 0.87–0.94) in HSIL, with a significant difference between groups (*P* = 0.0289). 5-ALA PDT for cervical SIL improves ORR and HPV clearance compared with observation and ablation, with efficacy comparable to conization and only mild adverse events. It is effective across lesion grades, with slightly better responses in HSIL.

**Supplementary Information:**

The online version contains supplementary material available at 10.1007/s10103-026-04955-9.

## Introduction

Cervical cancer ranks fourth in both incidence and mortality among women worldwide, with approximately 660,000 new cases and 350,000 deaths annually [1]. Persistent infection with high-risk human papillomavirus (HR-HPV), particularly human papillomavirus (HPV) types 16 and 18, is the primary etiological factor responsible for the development and progression of cervical lesions [2]. From HPV infection to cervical cancer typically takes several decades. Timely intervention at the early stage of the lesion can not only significantly improve patients’ clinical prognosis but also effectively prevent the progression of the lesion toward cervical cancer.

According to the fifth edition of the World Health Organization Classification of Tumors of the Female Reproductive Organs [3], squamous intraepithelial lesions(SIL) are classified into two categories based on the degree of epithelial abnormality: low-grade squamous intraepithelial lesion (LSIL) and high-grade squamous intraepithelial lesion (HSIL). Studies have shown that approximately 60% of LSIL lesions regress spontaneously [4]. Therefore, LSIL is generally managed through observation and regular follow-up, while patients at higher risk of progression to HSIL are managed according to colposcopy and diagnostic conization when indicated [5]. HSIL is considered a precancerous lesion of cervical cancer and carries a substantially higher risk of progression than LSIL. Studies have shown that approximately 20–30% of HSIL cases may progress to invasive cervical cancer within ten years. Therefore, current guidelines recommend stratified management for HSIL, with treatment for most patients and observation for selected CIN2 cases to reduce progression risk while avoiding overtreatment [6]. The main treatment approaches include cervical conization and ablation [7]. However, conization may disrupt cervical anatomy and integrity, leading to cervical insufficiency and an increased risks of miscarriage and preterm birth, while ablation therapy may be unable to precisely target dysplastic cells and is associated with relatively high recurrence rates [8].

Photodynamic therapy (PDT) is a novel minimally invasive technique characterized by minimal tissue damage, mild adverse effects, and rapid functional recovery. In recent years, PDT has been increasingly applied to the treatment of lower genital tract diseases. The therapeutic effect of PDT relies on three key elements: a photosensitizer, light irradiation at a specific wavelength, and the presence of molecular oxygen. 5-Aminolevulinic acid (5-ALA) is a commonly used second-generation photosensitizer precursor that can be selectively taken up by proliferating abnormal epithelial cells and converted into protoporphyrin IX (PpIX). Upon activation by light of a specific wavelength, PpIX transfers energy to surrounding oxygen molecules, producing reactive oxygen species (ROS) that induce oxidative damage, apoptosis, and necrosis of target cells [9, 10]. These properties support the potential role of 5-ALA PDT as a fertility-preserving treatment option for women of reproductive age with cervical lesions.

In recent years, growing attention has been paid to the therapeutic potential of 5-ALA PDT for cervical squamous intraepithelial lesion. However, existing studies are limited by small sample sizes and variability in the reported efficacy and safety outcomes. The present study aims to systematically review and quantitatively evaluate the therapeutic efficacy and safety of 5-ALA PDT compared with conventional treatments in patients with cervical SIL, providing evidence-based guidance for clinical decision-making.

## Materials and methods

### Protocol and guidance

This study was conducted in compliance with the PRISMA statement, and the protocol for this review was registered in PROSPERO (CRD420251122208).

### Inclusion criteria

Studies eligible for inclusion had to meet the following criteria:(1) population: women with histologically confirmed cervical squamous intraepithelial lesion (LSIL and HSIL). (2) Intervention: treatment with 5-ALA-PDT, without restrictions on photosensitizer concentration, wavelength of the light source, or other treatment parameters. (3) comparator: observation, ablation or conization. (4) outcomes: reporting at least one of the following outcomes: overall response rate or HPV clearance rate. (5) follow-up: a minimum follow-up period of six months.

### Exclusion criteria

The following studies were excluded: (1) studies without a comparator group. (2) studies in which PDT was combined with other therapies. (3) studies involving patients with recurrent lesion after prior surgical treatment. (4) review articles, conference abstracts, case reports, animal or in vitro studies. (5) studies lacking sufficient extractable outcome data. (6) duplicate publications.

### Outcomes

The primary outcome was the overall response rate (ORR). ORR was defined as the proportion of patients achieving complete or partial remission of cervical SIL at the final follow-up, as determined by histopathological findings. Secondary outcomes included complete remission (CR) rate, HPV clearance rate, ORR stratified by lesion extent, progression rate, recurrence rate and incidence of adverse events.

### Search strategy

A comprehensive literature search was conducted in both international and Chinese databases, including PubMed, Embase, the Cochrane Library, China National Knowledge Infrastructure, the Chinese Biomedical Literature Database and VIP Database. The search covered all records from database inception to 7 October 2025. We also searched ClinicalTrials.gov and ChiCTR.org to identify ongoing or unpublished eligible trials. The comprehensive search strategies of PubMed and Embase are explicitly presented in Table S1, and this strategy was adapted for other databases.

### Study selection

All retrieved records were imported into EndNote for reference management, and duplicate studies were removed. Two reviewers (W.G. and R.H.) then independently screened the titles and abstracts, followed by full-text assessment of potentially eligible articles. Final inclusion was determined according to the predefined eligibility criteria. Any disagreements between the two reviewers were resolved through discussion or adjudication by a third reviewer (Y.H.).

### Data collection process

Data were extracted by one reviewer (W.G.) and independently verified by another reviewer (R.H.), with any discrepancies resolved through discussion to reach consensus. The extracted information included the following: author, year of publication, study design, sample size, type of HPV infection, photodynamic therapy parameters, comparator interventions, follow-up duration, ORR, CR rate, HPV clearance rate, ORR stratified by lesion extent, progression rate, recurrence rate, and occurrence of adverse events.

### Assessment of risk of bias and quality of evidence

Two researchers (W.G. and K.G.) independently assessed the methodological quality of all included studies. Methodological quality and risk of bias of non-randomized controlled trial (NRCT) studies were assessed using the ROBINS-I tool [11], and ROB2 tool [12] was used for randomized controlled trial (RCT) studies. The quality of evidence was performed using the GRADEpro [13].

### Data synthesis

We performed statistical analyses using the meta package in R (version 4.4.3). Effect sizes were estimated using odds ratios (OR) with 95% confidence intervals (CI), and heterogeneity was assessed using the I^2^ test [14]. Random-effects models were applied due to methodological heterogeneity and the small number of studies, to reduce the risk of type I error [15]. Potential publication bias and small-study effects were assessed with funnel plots and Egger’s test, and sensitivity analyses were performed to examine the robustness of the pooled estimates [16]. Besides, publication bias was not assessed when fewer than five studies were available for an outcome due to limited statistical power. Because this study compared 5-ALA PDT with different treatments, we performed subgroup analyses according to the type of comparator: observation, ablation and conization. In addition, single-arm analyses were conducted to evaluate the efficacy of 5-ALA PDT between LSIL and HSIL. These analyses were based on logit-transformed proportions and fitted using generalized linear mixed models. Only outcomes supported by data from at least two independent studies were included in the main analysis, in order to avoid presenting potentially unreliable evidence where data synthesis was not feasible.

## Results

### Eligible studies and study characteristics

**Fig. 1:**
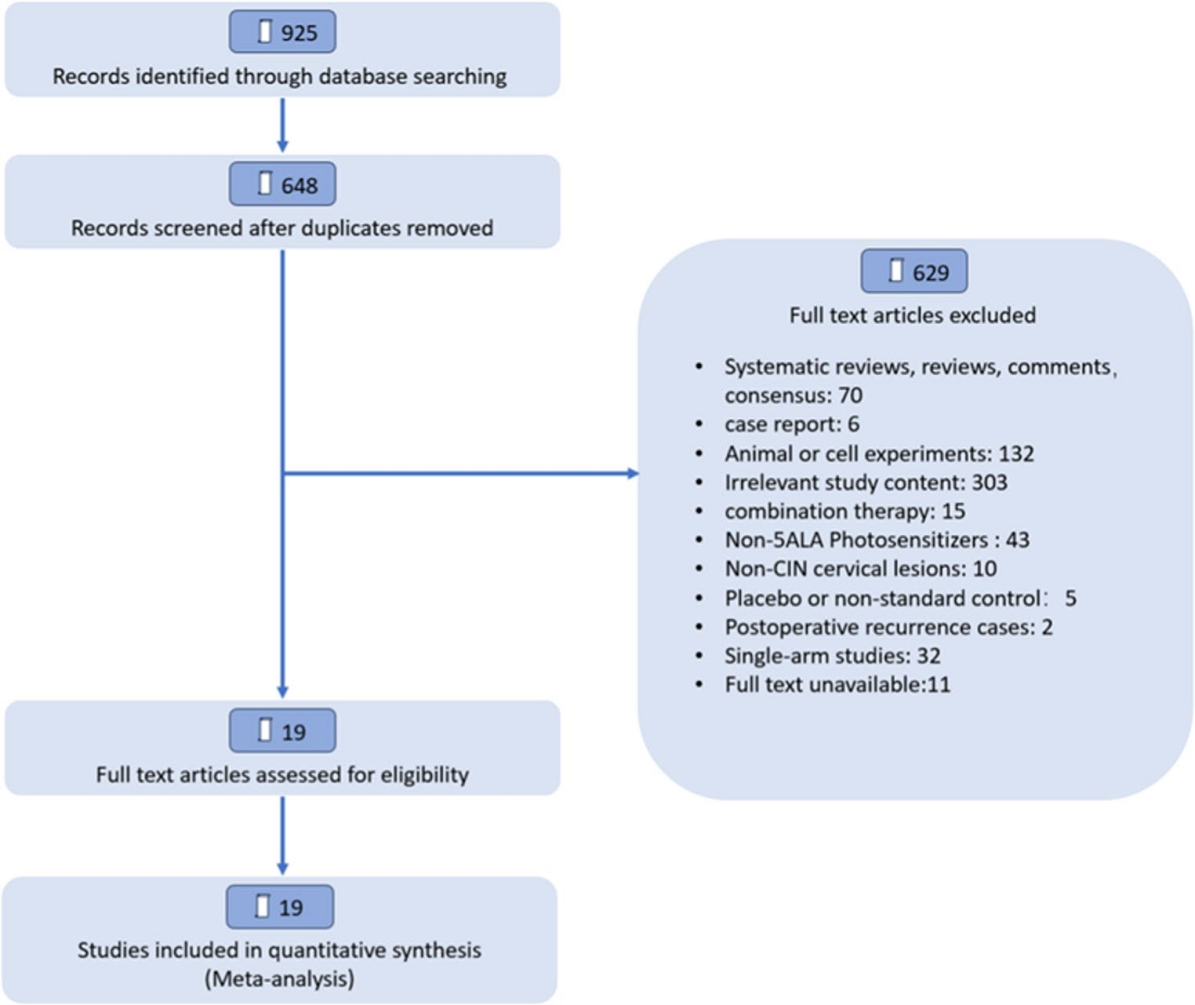
Search strategy and final included and excluded studies

A total of 925 records were initially identified. After removing duplicates and excluding studies that did not meet the inclusion and exclusion criteria, 18 NRCT [17–34]studies and 1 RCT study [35] were finally included in this review (Fig. 1). A total of 3,354 patients were included across all studies, comprising 1,642 patients in the 5-ALA PDT group, 637 in the observation group, 470 in the ablation group, and 535 in the conization group. All observation cohorts were restricted to LSIL populations, all conization cohorts were limited to HSIL populations, whereas ablation therapy studies enrolled a mixed population of LSIL and HSIL patients. Among the included studies, 7 were judged to have a low risk of bias and 12 were rated as having a moderate risk of bias. Table 1 presents the summary characteristics of included studies. Table S2 provides detailed study characteristics. Table S3 shows the detailed risk of bias for NRCT study as assessed using the ROBINS-I tool, while Table S4 shows the detailed quality assessment of the RCT study.

**Table 1:** Summary characteristics of included studies

Characteristics	No. of studies (No. of patients)
Study design:	
NRCT	18 (3221)
RCT	1 (133)
Country:	
China	18 (3332)
Austria	1 (22)
Lesion severity:	
LSIL	9 (1886)
HSIL	10 (1468)
Intervention*:	
5-ALA PDT	19 (1642)
Observation	6 (637)
Ablation	5 (470)
Conization	9 (535)

* One included study had control groups comprising two treatment modalities: ablation and conization

### Overall response rate

**Fig. 2:**
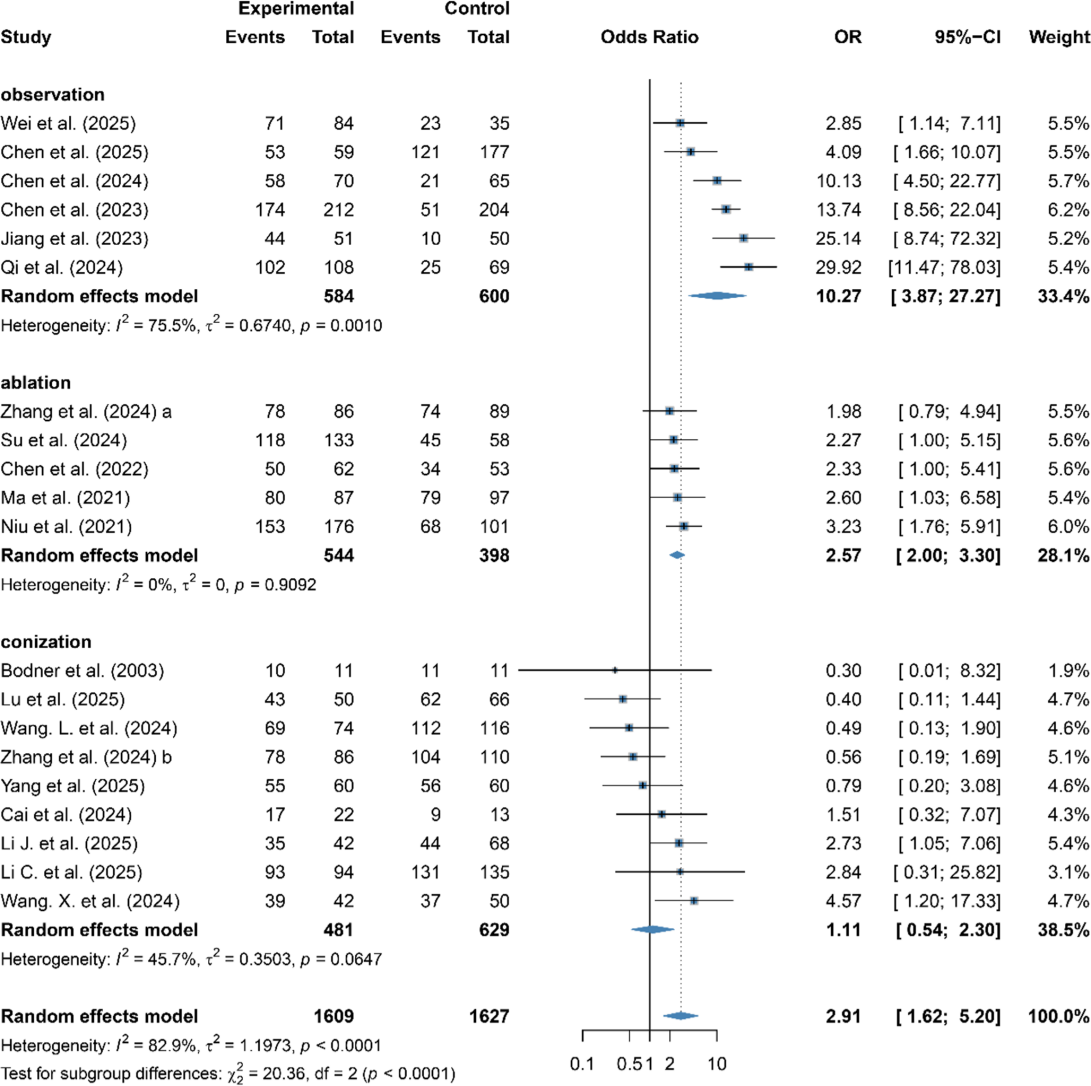
Forest plot comparing the ORR between 5-ALA PDT and control treatments

All 19 studies reported ORR results (Fig. 2). Because pooling different comparator treatments resulted in substantial heterogeneity and limited clinical comparability, we focused on subgroup analyses comparing PDT with each specific treatment modality. Compared with observation, 5-ALA PDT significantly improved ORR in the observation group (OR = 10.27, 95% CI: 3.87–27.27, I^2^ = 75.5%). Sensitivity analysis (Fig. S1A) suggested that Wei et al. (2025) partly contributed to heterogeneity. Subgroup analyses also suggested that differences in the number of treatment sessions may contribute to the observed heterogeneity in ORR (Fig. S2). PDT also showed superior efficacy to ablation (OR = 2.57, 95% CI: 2.00-3.30, I^2^ = 0%), while no significant advantage was observed over conization (OR = 1.11, 95% CI: 0.54–2.30, I^2^ = 45.7%). We further performed a lesion-grade stratified analysis for ORR (Fig. S3). Among LSIL patients, 5-ALA PDT was associated with a higher ORR than observation (OR = 10.27, 95% CI:3.87–27.27) and ablation (OR = 2.71, 95% CI: 1.62–4.55). No significant difference in ORR was observed between PDT and ablation (OR = 2.26, 95% CI: 0.39–13.06) or conization (OR = 1.11, 95% CI: 0.54–2.30) in HSIL patients. This finding suggests that 5-ALA PDT was associated with higher ORR than observation and potential advantage over ablation, particularly in LSIL patients, while achieving a response comparable to conization.

Funnel plot (Fig. S4) analysis revealed no apparent asymmetry in any subgroup, and the Egger’s test (Table S5) detected no significant small-study effects or publication bias.

### Complete remission rate

**Fig. 3:**
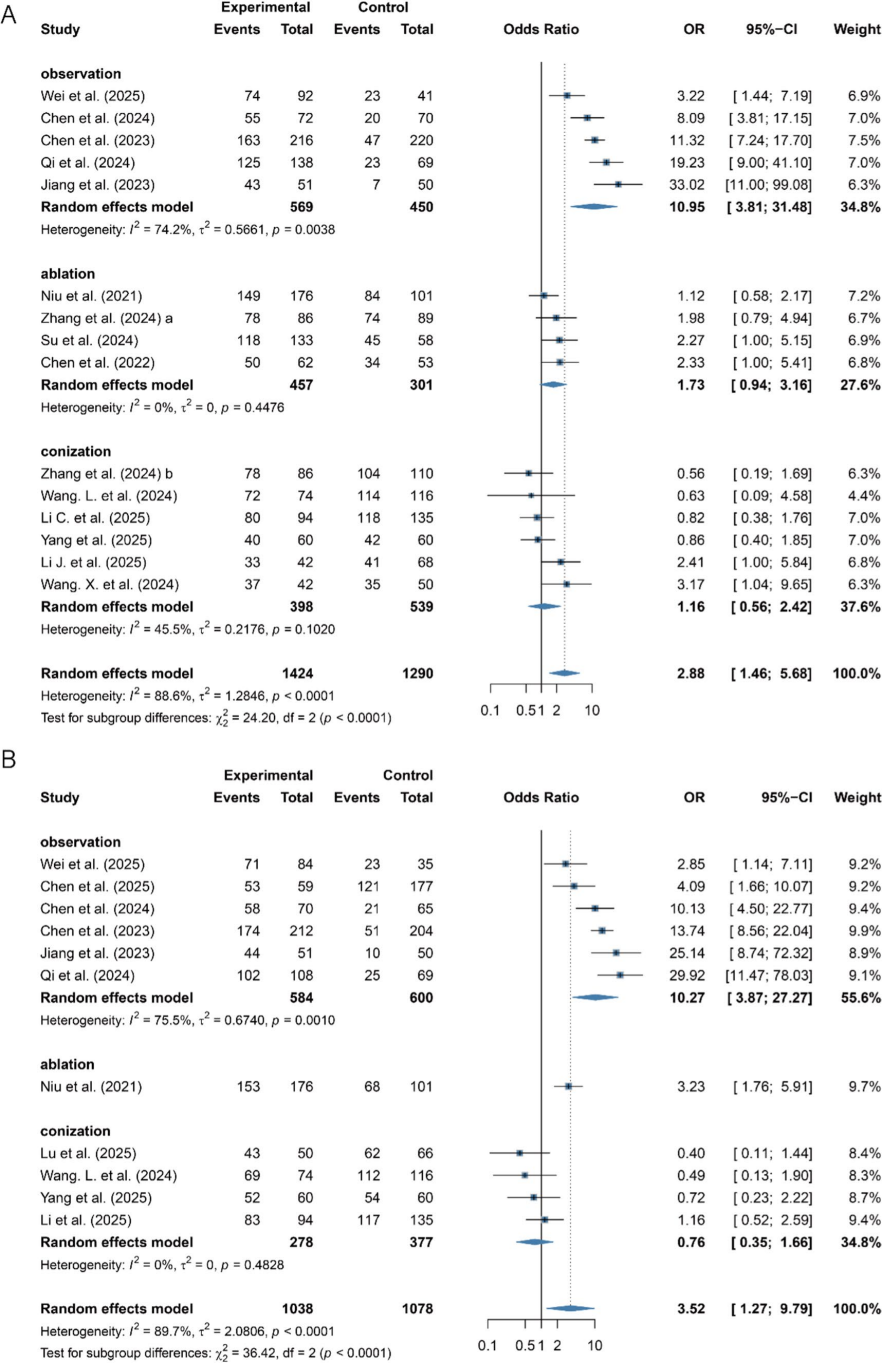
Forest plot comparing the CR rate between 5-ALA PDT and control treatments: **A** 6-month follow-up; **B** 12-month follow-up

14 studies reported the 6-month CR rate (Fig. 3A). The forest plot demonstrated that the CR rate after PDT was significantly higher than observation group (OR = 10.95, 95% CI: 3.81–31.48, I^2^ = 74.2%). Although PDT also showed a higher CR rate than ablation, the difference was not statistically significant (OR = 1.73, 95% CI: 0.94–3.16, I^2^ = 0%). Compared with conization, no significant difference in CR rate was observed (OR = 1.16, 95% CI: 0.56–2.42, I^2^ = 45.5%). The supplementary lesion-grade stratified analysis showed a similar result for 6-month CR rate (Fig. S5). At the 12-month follow-up, 10 studies were included (Fig. 3B), the forest plot showed that the CR rate of PDT was significantly higher than the observation group (OR = 10.27, 95% CI: 3.87–27.27, I2 = 75.5%). In contrast, no significant difference in CR rate was observed between PDT and conization (OR = 0.76, 95% CI: 0.35–1.66, I^2^ = 0%). High heterogeneity was observed within the observation subgroup at both 6- and 12-month follow-ups. Sensitivity analyses suggested that this heterogeneity may be partly related to Wei *et al. *(2025), although it was not fully explained by this study alone (Fig. S1B and S1C). These results indicate that 5-ALA PDT may promote histological remission compared with observation, whereas its advantage over ablation or conization remains less certain.

### HPV clearance rate

**Fig. 4:**
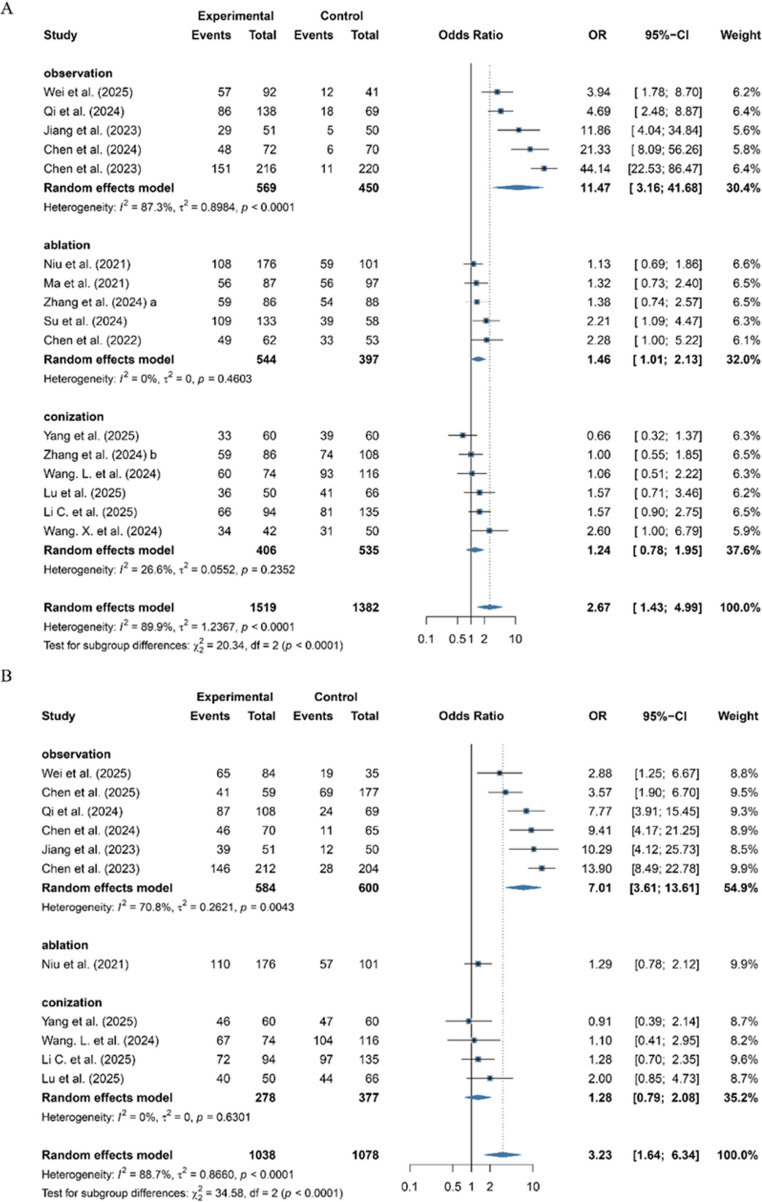
Forest plot comparing the HPV clearance rate between 5-ALA PDT and control treatments: **A** 6-month follow-up; **B** 12-month follow-up

A total of fifteen studies evaluated the HPV clearance rate at the 6-month follow-up (Fig. 4A). Forest plot indicated that 5-ALA PDT achieved a significantly higher HPV clearance rate than observation (OR = 11.47, 95% CI: 3.16–41.68, I^2^ = 87.3%). Compared with ablation, PDT was associated with a modestly higher HPV clearance rate (OR = 1.46, 95% CI: 1.01–2.13, I^2^ = 0%), while no significant difference was observed between PDT and conization (OR = 1.24, 95% CI: 0.78–1.95, I2 = 26.6%). The lesion-grade stratified analysis (Fig. S6) showed a generally similar pattern. PDT was associated with a higher clearance rate than observation in LSIL patients, with no significant difference versus conization in HSIL patients and only a modest advantage over ablation in HSIL patients.

At the 12-month follow-up, eleven studies reported HPV clearance rate (Fig. 4B). PDT markedly improved HPV clearance rate compared with observation (OR = 7.01, 95% CI: 3.61–13.61, I^2^ = 70.8%). The clearance rate after PDT was also higher than conization, though the difference was not statistically significant (OR = 1.28, 95% CI: 0.79–2.08, I² = 0%). The observation subgroup exhibited high heterogeneity at both 6- and 12-month follow-ups, sensitivity analyses (Fig. S1D and S1E) suggested that this heterogeneity may be partly related to Chen et al. (2023). Subgroup analyses indicated that treatment session frequency can also partly explain the heterogeneity observed in 12-month HPV clearance (Fig. S7). The improved HPV clearance observed with PDT may be clinically relevant because persistent HR-HPV infection is a key driver of lesion persistence and progression.

We also analyzed the clearance rate of HPV 16/18 (Fig. S8). Three studies in the observation subgroup reported this outcome, suggesting a higher HPV16/18 clearance rate after PDT than after observation (OR = 7.21, 95% CI: 2.83–18.39, I^2^ = 0%). However, this exploratory finding should be interpreted cautiously because it was based on only three studies and the confidence interval was wide.

### ORR stratified by lesion extent

Two studies reported treatment outcomes in lesions involving the cervical canal (Fig. S9A). The ORR of 5-ALA PDT in this condition was significantly lower than other treatment (OR = 0.16, 95% CI: 0.06–0.46, I^2^ = 0%). Two studies reported outcomes in lesions with gland involvement (Fig. S9B). Compared with conization, PDT did not show a therapeutic advantage in these cases (OR = 0.50, 95% CI: 0.37–0.66, I^2^= 0%). Because only two studies informed each analysis, these findings should be regarded as exploratory and interpreted cautiously until confirmed in larger studies.

### Recurrence rate

A total of ten studies reported lesion recurrence rates after treatment. Two studies in which both the PDT and control groups reported zero recurrence events were excluded. The remaining eight studies were included in this analysis (Fig. S10). Subgroup analysis revealed that PDT was associated with a significantly lower risk of lesion recurrence compared with ablation (OR = 0.30, 95% CI: 0.14–0.63, I^2^ = 0%), whereas no significant difference was found when PDT was compared with observation (OR = 0.71, 95% CI: 0.00-249.02) or conization (OR = 1.47, 95% CI: 0.35–6.22). The recurrence result in the observation subgroup should be regarded as exploratory because sparse events and few studies led to a very wide confidence interval.

### Progression rate

The progression rate after PDT was significantly lower than that in the observation group (OR = 0.19, 95% CI: 0.11–0.34, I^2^ = 0%), and although PDT also showed an advantage compared with the ablation group (OR = 0.67, 95% CI: 0.30–1.54, I2 = 0%), the difference was not statistically significant (Fig. S11).

### Adverse events

Adverse event is an important indicator for evaluating treatment safety. Owing to the low incidence of adverse events and the resulting instability and limited interpretability of pooled effect estimates, adverse outcomes in this study were summarized in tabular form for descriptive comparison (Table 2) and detail information can be seen in Table S6.

Common adverse reactions after PDT included pain, increased vaginal secretions, pruritus, burning, and bleeding, while some studies also reported lower abdominal distension and dysmenorrhea. The adverse events associated with 5-ALA PDT were generally mild and well tolerated. The incidence of pruritus and burning was higher in the PDT group compared with other treatments, whereas most other adverse reactions tended to occur less frequently in the PDT group. In addition, ulceration, infection, cervical canal adhesion, cervical shortening, scarring, malodorous discharge, and obstructed menstrual flow were reported in the ablation or conization groups but were not observed in the PDT group.

**Table 2:** Adverse events

Adverse event	Control intervention	No. of studies	PDT, n/N (%)	Control, n/N (%)
Pain	Ablation	2	24/220 (10.91%)	26/155 (16.77%)
	Conization	3	24/152 (15.79%)	65/176 (36.93%)
Increased vaginal secretions	Ablation	2	28/220 (12.73%)	102/155 (65.81%)
	Conization	3	66/184 (35.87%)	102/242 (42.15%)
Pruritus	Ablation	2	9/220 (4.09%)	6/155 (3.87%)
	Conization	1	7/74 (15.91%)	4/116 (3.45%)
Bleeding	Ablation	4	6/368 (1.63%)	17/318 (5.34%)
	Conization	5	5/312 (1.60%)	87/381 (22.83%)
Burning	Conization	1	9/74 (12.16%)	13/116 (11.21%)
Abdominal distension	Conization	1	16/72 (22.22%)	28/76 (36.94%)
Dysmenorrhea	Ablation	1	1/62 (1.61%)	1/53 (1.89%)

### Subgroup analysis by lesion grade

**Fig. 5:**
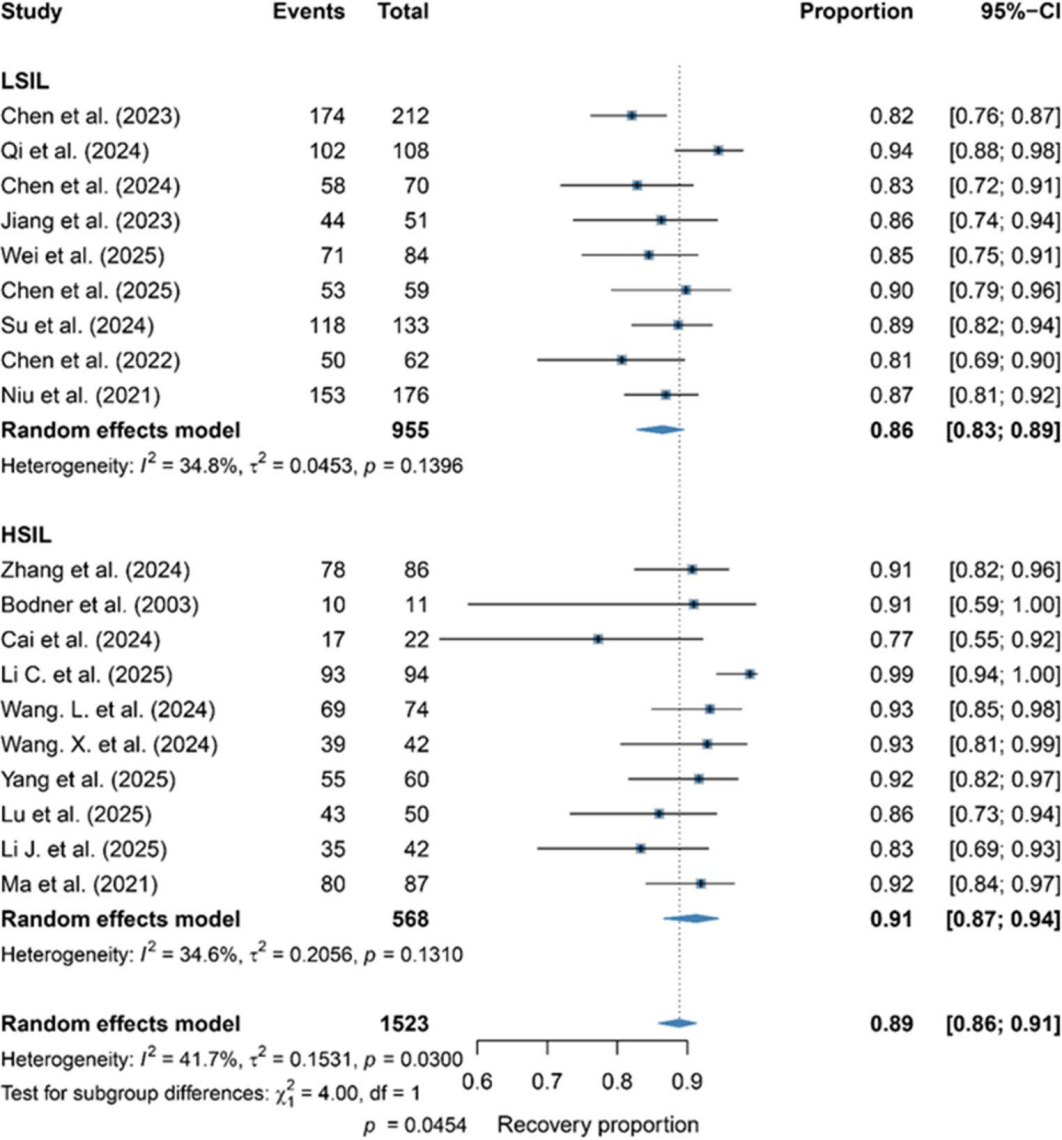
Forest plot of ORR after 5-ALA PDT in patients with different lesion grades

To further explore the clinical efficacy of 5-ALA PDT between LSIL and HSIL, we conducted single-arm meta-analyses stratified by lesion severity (Fig. 5). The pooled ORR was 0.86 (95% CI: 0.83–0.89) in LSIL and 0.91 (95% CI: 0.87–0.94) in HSIL, with a statistically significant subgroup difference (*P* = 0.0454). Notably, treatment frequency varied across studies, with LSIL predominantly treated with three sessions and HSIL with six sessions. Further analysis in Fig. S12 showed no significant difference in ORR between three and six treatment sessions in either LSIL (*P* = 0.5384) or HSIL (*P* = 0.7618).

However, the 12-month CR rate was similar between the two groups (Fig. S13): 0.87 (95% CI: 0.82–0.90) in the LSIL group and 0.89 (95% CI: 0.81–0.94) in the HSIL group, with no statistically significant difference (*P* = 0.4154). Regarding HPV clearance at 12-month follow-up (Fig. S14), PDT achieved 0.71 (95% CI: 0.65–0.77) clearance in LSIL and 0.81(95% CI: 0.69–0.89) in HSIL, showing a modest but significant difference in favor of HSIL (*P* = 0.0201).

### GRADE assessment

The GRADE assessment of the outcomes is presented in Table S7. Overall, the certainty of evidence was low to very low for most outcomes, mainly due to the predominance of non-randomized studies, inconsistency, imprecision, or sparse events. This suggests that the true effects may differ from the pooled estimates, and future high-quality studies could change the direction or magnitude of the observed effects.

### Clinical applicability

5-ALA PDT causes limited damage to cervical structure and function and may be suitable for carefully selected women who wish to preserve reproductive potential. According to expert consensus [36], potential candidates include patients with histologically confirmed LSIL/CIN1 and selected HSIL/CIN2 patients with a fully visible squamocolumnar junction and upper lesion margin on colposcopy. ALA-PDT is not recommended when atypical glandular cells, adenocarcinoma in situ, suspected malignancy, or possible malignancy cannot be excluded. Wang et al*.* (2024) [20] provided representative pre- and post-treatment colposcopic and histopathological images in the article, offering a practical example of the PDT treatment course.

## Discussion

This study systematically evaluated the efficacy and safety of 5-ALA PDT in the treatment of cervical SIL.The results showed that 5-ALA PDT was associated with higher ORR and CR rates than observation group. Compared with ablation, PDT showed a higher ORR mainly in LSIL patients, while no clear advantage was observed for 6-month CR rate within either lesion-grade subgroup. No significant difference was observed between PDT and conization, which is consistent with previous systematic reviews of surgical management [37, 38]. Mechanistically, 5-ALA PDT selectively accumulates PpIX within dysplastic epithelium and, upon light activation, generates reactive oxygen species that preferentially ablate lesional cells and occlude abnormal microvasculature while sparing surrounding normal tissue [10, 39]. In addition to its direct cytotoxic effects, PDT may also modulate the local immune microenvironment, promoting CD8^+^T cell infiltration [40] and dendritic cell maturation [41], thereby contributing to tumor suppression and viral clearance through multiple mechanisms.

We included 19 studies, of which only one RCT; the remainder were NRCTs. Because treatment allocation in most included studies was not randomized, selection bias and confounding by indication cannot be excluded. The observed between-group differences should therefore be interpreted as associations rather than definitive causal effects, and the effects of 5-ALA PDT may have been overestimated. The GRADE assessment showed low to very low certainty for most outcomes, indicating that future high-quality studies may change the magnitude or direction of the observed effects. Therefore, the current findings should be interpreted cautiously and confirmed in well-designed randomized controlled trials.

High heterogeneity was observed across multiple outcomes, limiting the robustness and generalizability of the pooled estimates. To address this, we conducted subgroup analyses by comparator type using random-effects models and reported prediction intervals. Sensitivity analyses and Egger’s tests were also performed to enhance the reliability of the inferences. Although sensitivity analyses identified Wei et al*. *(2025) and Chen et al*. *(2023) as contributing to heterogeneity in some outcomes, heterogeneity could not be explained by a single study alone. To explore potential sources, we assessed variables including PDT parameters, lesion characteristics, HPV genotype distribution, and treatment sessions et al. Most variables showed minor differences or were incompletely reported (Table S8). Treatment sessions may partly explain heterogeneity in ORR and 12-month HPV clearance, but each subgroup included only two studies and the confidence intervals were wide, indicating low precision. Overall, while exploratory analyses provide insight into potential heterogeneity sources, pooled estimates and subgroup findings should be interpreted cautiously, and further well-designed randomized trials are warranted.

Among the 19 included studies, 18 were conducted in China and only one in Austria. The evidence base was therefore derived almost entirely from Chinese populations, which may limit generalizability. High-risk HPV genotype distributions differ across regions. HPV52 and HPV58 are relatively common in China and other Asian populations, whereas HPV18 and HPV45 are more frequently reported in Europe and the United States [42]. Because HPV genotype may influence viral persistence, lesion progression, and treatment response, HPV clearance results should be interpreted cautiously across populations. In addition, the 5-ALA products, treatment parameters, and clinical protocols used in the included studies mainly reflected Chinese clinical practice [43]. Together with differences in screening, referral pathways, and healthcare systems, direct extrapolation to FDA- or EMA-regulated settings may not be fully appropriate.

Because this review compares PDT with three recommended approaches, the enrolled populations differed by comparator: all observation cohorts comprised LSIL, all conization cohorts comprised HSIL, and ablation group included both HSIL and LSIL. This structural imbalance in baseline lesion severity represents a major source of bias and may affect the interpretation of the pooled effect estimates. Differences in outcomes may partly reflect baseline disease severity rather than the treatment effect alone. To further examine this issue, we performed supplementary analyses stratified by lesion grade for the main outcomes. These analyses showed that the difference between PDT and ablation in ORR varied across lesion grades. In contrast, the patterns for the 6-month CR rate and HPV clearance were generally consistent with the main analysis. Therefore, although the overall findings suggest potential benefits of 5-ALA PDT, the conclusions should be interpreted cautiously in light of this structural imbalance.

Compare with observation group, PDT significantly increases lesion regression and HPV clearance compared with observation, and it helps prevent disease progression. Our exploratory findings also suggest a potential benefit of 5-ALA PDT for HPV16/18 clearance, although the evidence remains limited by the small number of studies. For lesions involving the cervical canal or glandular structures, current limited evidence suggests that PDT may be less effective than standard therapies. Both conclusions rest on a small evidence base with substantial uncertainty, so they should be interpreted cautiously. Rigorous prospective comparative studies are needed to confirm these findings.

Notably, although HPV clearance is an important endpoint, it should be understood as HPV DNA negativity, or apparent clearance, rather than complete viral eradication. Patients may remain in a state of subclinical persistence. In this state, the virus may persist in epithelial basal cells under immune control, with low-level viral gene expression or occasional virion production, while HPV DNA remains below the clinical detection threshold [44, 45]. When immune surveillance weakens, viral expression may increase again and lead to HPV redetection. Because cancer prevention is more likely to depend on sustained viral control rather than transient HPV negativity, patients who achieve short-term HPV clearance after 5-ALA PDT may still have a risk of subsequent HPV positivity, and regular follow-up remains necessary after treatment.

5-ALA PDT demonstrates favorable efficacy in both LSIL and HSIL patients. The ORR and CR rate exceeded 0.85 in both groups, and the 12-month HPV clearance rates were 0.71 and 0.81, respectively. These findings indicate that PDT effectively promotes lesion regression and HPV clearance across different grades of cervical lesions. Notably, the ORR and 12-month HPV clearance rate were significantly higher in HSIL than in LSIL, whereas the 12-month CR rate showed no significant difference between the two groups. This pattern may be attributed to the biological characteristics of HSIL lesions, which are characterized by higher cellular proliferation and metabolic activity, resulting in greater PpIX accumulation and stronger photodynamic cytotoxicity. It should be noted that ORR includes both partial and complete responses. In HSIL patients, histopathological downgrading is also considered a treatment response, which contributes to a higher ORR. In contrast, the CR rate represents a more stringent endpoint, requiring complete normalization of the cervical epithelium. As a result, the difference between LSIL and HSIL is attenuated when assessed using CR as the outcome measure.

In summary, 5-ALA PDT offers an effective treatment option for both LSIL and HSIL patients. However, given the limitations of the current evidence, larger and methodologically rigorous studies are needed to more precisely define its efficacy, optimize treatment protocols, and identify the most suitable patient populations. Future studies should also incorporate patient-reported outcome measures, including procedural pain, psychological stress, treatment satisfaction, and quality of life. These outcomes have been increasingly used to assess recovery and patient-centered benefits after minimally invasive procedures [46].

## Conclusions

In summary, 5-ALA PDT was associated with improved lesion regression and HPV clearance compared with observation in patients with LSIL, with potential benefits in reducing progression. Compared with ablation, PDT showed favorable effects on ORR, and HPV clearance, although the magnitude of benefit varied by lesion grade. It also showed comparable efficacy to conization, while potentially causing less cervical tissue damage and fewer adverse events. Subgroup analyses further demonstrated that 5-ALA PDT is highly effective across lesion grades, with HSIL showing higher ORR and HPV clearance rates than LSIL. 5-ALA PDT represents a promising treatment option for appropriately selected patients with cervical SIL.

## Supplementary Information

Below is the link to the electronic supplementary material.

Supplementary Material 1

## Data Availability

No new data were created in this study.
