# Supplementary material for: Efficacy and safety of 5-aminolevulinic acid photodynamic therapy for cervical squamous intraepithelial lesion: a systematic review and meta-analysis
Source: Lasers Med Sci. 2026 Jul 18;41(1):154. doi: 10.1007/s10103-026-04955-9 (PMC13380584; doi:10.1007/s10103-026-04955-9)
Supplement: Supplementary file 1 — Supplementary Material 1 [file 10103_2026_4955_MOESM1_ESM.docx]

**Efficacy and safety of 5-aminolevulinic acid photodynamic therapy for cervical squamous intraepithelial lesion: a systematic review and meta-analysis**

**Table S1: Search strategy**

| **PubMed** | | |
| --- | --- | --- |
| #1 | ("Photochemotherapy"[MeSH] OR "PDT"[TW] OR "Photodynamic Therap*"[TW] OR "ALA-PDT"[TW] OR "5-ALA PDT"[TW] OR "5-aminolevulinic acid photodynamic therap*"[TW] OR "5-aminolevulinic acid*"[TW]) | 47,058 |
| #2 | ("Uterine Cervical Neoplasms"[MeSH] "Squamous Intraepithelial Lesions"[MeSH] OR "LSIL"[TW] OR "HSIL"[TW] OR "CIN"[TW] OR "Cervical Neoplas*"[TW]) | 101,372 |
| #3 | #1 AND #2 | 313 |
| **Embase** | | |
| #1 | 'uterine cervix dysplasia'/exp | 8,648 |
| #2 | 'dysplasia of the uterine cervix':ab,ti OR 'uterine cervix squamous dysplasia':ab,ti OR 'uterine cervix dysplasia':ab,ti OR 'uterine cervical dysplasia':ab,ti OR 'cervical dysplasia,uterine':ab,ti OR 'dysplasia, uterine cervical':ab,ti OR 'cervical dysplasia':ab,ti OR'cervical dysplasias':ab,ti OR 'dysplasia, cervical':ab,ti OR 'dysplasia of cervix uteri':ab,ti OR 'cervix uteri dysplasia':ab,ti OR 'cervix uteri dysplasias':ab,ti OR 'cervix dysplasia':ab,ti OR 'dysplasia, cervix':ab,ti OR 'cervical intraepithelial neoplasms':ab,ti OR'cervical intraepithelial neoplasm':ab,ti OR'intraepithelial neoplasm, cervical':ab,ti OR 'intraepithelial neoplasms, cervical':ab,ti OR 'neoplasm, cervical intraepithelial':ab,ti OR 'neoplasms, cervical intraepithelial':ab,ti OR 'intraepithelial neoplasia, cervical':ab,ti OR 'neoplasia, cervical intraepithelial':ab,ti OR 'cervical intraepithelial neoplasia':ab,ti OR'cervical intraepithelial neoplasia, grade iii':ab,ti | 15,520 |
| #3 | 'squamous intraepithelial lesion of the cervix'/exp | 1,856 |
| #4 | 'squamous intraepithelial lesions':ab,ti OR'intraepithelial lesion, squamous':ab,ti OR'lesions, squamous intraepithelial':ab,ti OR'squamous intraepithelial lesion':ab,ti OR'low-grade squamous intraepithelial lesions':ab,ti OR 'low grade squamous intraepithelial lesions':ab,ti OR 'lsil, low-grade squamous intraepithelial lesion':ab,ti OR 'lsil, low grade squamous intraepithelial lesion':ab,ti OR 'lsil, low-grade squamous intraepithelial lesions':ab,ti OR 'lsil, low grade squamous intraepithelial lesions':ab,ti OR 'low-grade squamous intraepithelial lesion':ab,ti OR 'low grade squamous intraepithelial lesion':ab,ti OR 'high-grade squamous intraepithelial lesions':ab,ti OR 'high grade squamous intraepithelial lesions':ab,ti OR 'hsil, high grade squamous intraepithelial lesion':ab,ti  OR 'high-grade squamous intraepithelial lesion':ab,ti OR 'high grade squamous intraepithelial lesion':ab,ti OR 'hsil, high-grade squamous intraepithelial lesions':ab,ti OR 'hsil, high grade squamous  intraepithelial lesions':ab,ti OR 'lsil, atypical squamous cells cannot exclude hsil':ab,ti OR 'lsil asc-h':ab,ti OR 'squamous intraepithelial cervical lesion':ab,ti OR 'squamous intraepithelial lesions of the cervix':ab,ti OR 'squamous intraepithelial lesion of the cervix':ab,ti | 7,596 |
| #5 | #1 OR #2 OR #3 OR #4 | 24,911 |
| #6 | 'photochemotherapy'/exp | 63,105 |
| #7 | 'photochemotherapies':ab,ti OR 'therapy, photodynamic':ab,ti OR 'photodynamic therapies':ab,ti OR 'therapies, photodynamic':ab,ti OR 'photodynamic therapy':ab,ti OR 'red light photodynamic therapy':ab,ti OR 'red light pdt':ab,ti OR 'light pdt, red':ab,ti OR 'pdt, red light':ab,ti OR 'ala-pdt':ab,ti OR '5-aminolevulinic acid photodynamic therapy':ab,ti OR '5-aminolevulinic acid':ab,ti OR 'chemophototherapy':ab,ti OR 'hematoporphyrin photoradiation':ab,ti OR 'photo-activated chemotherapy':ab,ti OR'photo-chemotherapy':ab,ti OR 'photoactivated chemotherapy':ab,ti OR 'photochemotherapy':ab,ti | 43,257 |
| #8 | #6 OR #7 | 70,648 |
| #9 | #5 AND #8 | 228 |

**Table S2: Study characteristics**

| Studies | Country | Study design | Lesion severity | Age | Sample size | Control | Risk of bias |
| --- | --- | --- | --- | --- | --- | --- | --- |
| Su *et al.* (2024) | China | NRCT | LSIL | 25-55 | 200(136/64) | Ablation (CO₂ laser) | Moderate |
| Zhang *et al.* (2024) a | China | NRCT | HSIL | 33.22±8.36/ 34.07±8.84 | 175 (86/89) | Ablation | Low |
| Zhang *et al.* (2024) b | China | NRCT | HSIL | 33.22±8.36/ 35.45±8.80 | 196 (86/110) | Conization (CKC/LEEP) | Low |
| Chen *et al.* (2022) | China | NRCT | LSIL | 36.23 ± 2.69 / 35.71 ± 2.51 | 115 (62/53) | Ablation (CO₂ laser) | Low |
| Bodner *et al.* (2003) | Austria | NRCT | HISL | 28/29 | 22(11/11) | Conization (CKC) | Low |
| Cai *et al.* (2024) | China | NRCT | HSIL | 30-65 | 120 (60/60) | Conization (LEEP) | Moderate |
| Chen *et al.* (2023) | China | NRCT | LSIL | 31.30 ± 7.12 / 31.68 ± 7.16 | 436 (216/220) | Observation | Low |
| Li C. *et al.* (2025) | China | NRCT | HSIL | 29.12 ± 6.74 / 31.43 ± 5.00 | 229 (94/135) | Conization (LEEP) | Moderate |
| Ma *et al.* (2021) | China | NRCT | HSIL | 33/32 | 184 (87/97) | Ablation (Cryotherapy) | Moderate |
| Niu *et al.* (2021) | China | NRCT | LSIL | 35.3 / 33.3 | 297 (185/112) | Ablation (CO₂ laser) | Low |
| Qi *et al.* (2024) | China | NRCT | LSIL | 43.3 ± 11.65 / 42.7 ± 11.97 | 207 (138/69) | Observation | Moderate |
| Wang L. *et al.* (2024) | China | NRCT | HSIL | 31.1 ± 6.2 / 29.4 ± 6.0 | 190 (74/116) | Conization (LEEP) | Moderate |
| Wang X. *et al.* (2024) | China | NRCT | HSIL | 29.4/ 33.2 | 92 (42/50) | Conization (LEEP) | Moderate |
| Yang *et al.* (2025) | China | NRCT | HSIL | 29.78 ± 1.27 / 31.38 ± 1.16 | 120 (60/60) | Conization (LEEP) | Low |
| Chen *et al.* (2024) | China | NRCT | LSIL | 34.57 ± 5.66 /34.57 ± 4.98 | 142 (72/70) | Observation | Moderate |
| Jiang *et al.* (2023) | China | NRCT | LSIL | 35.45 /34.33 | 120 (60/60) | Observation | Moderate |
| Lu *et al.* (2025) | China | NRCT | HSIL | 28.36±5.38/30.02±4.52 | 116(50/66） | Conization (LEEP) | Low |
| Li J.*et al.* (2025) | China | NRCT | HSIL | 28.2 ± 6.1/32 | 110(42/68) | Conization (LEEP) | Moderate |
| Chen *et al.* (2025) | China | NRCT | LSIL | 34/38 | 236(59/177) | Observation | Moderate |
| Wei *et al.* (2025) | China | RCT | LSIL | 39.46±7.61/39.22±6.68 | 133(92/41) | Observation | Some concerns |

**Table S3 Quality assessment of the included NRCT studies**

| Studies | bias due to confounding | bias in classification of intervention | bias in selection of participants | bias due to deviations from intended intervention | bias due to missing data | bias arising from measurement of the outcome | bias in selection of the reported result |
| --- | --- | --- | --- | --- | --- | --- | --- |
| Su *et al.* (2024) | Low | Low | Low | Low | Moderate | Low | Low |
| Zhang *et al.* (2024) | Low | Low | Low | Low | Low | Low | Low |
| Chen *et al.* (2022) | Low | Low | Low | Low | Low | Low | Low |
| Bodner *et al.* (2003) | Low | Low | Low | Low | Low | Low | Low |
| Cai *et al.* (2024) | Moderate | Moderate | Moderate | Moderate | Moderate | Moderate | Low |
| Chen *et al.* (2023) | Low | Low | Low | Low | Low | Low | Low |
| Li *et al.* (2025) | Low | Low | Low | Low | Moderate | Low | Low |
| Ma *et al.* (2021) | Low | Low | Low | Low | Moderate | Low | Low |
| Niu *et al.* (2021) | Low | Low | Low | Low | Low | Low | Low |
| Qi *et al.* (2024) | Moderate | Low | Low | Low | Moderate | Low | Low |
| Wang L. *et al.* (2024) | Low | Low | Low | Low | Moderate | Low | Low |
| Wang X. *et al.* (2024) | Moderate | Moderate | Low | Low | Low | Low | Low |
| Yang *et al.* (2025) | Low | Low | Low | Low | Low | Low | Low |
| Chen *et al.* (2024) | Low | Moderate | Low | Low | Low | Low | Low |
| Jiang *et al.* (2023) | Moderate | Moderate | Low | Low | Moderate | Low | Low |
| Lu *et al.* (2025) | Low | Low | Low | Low | Low | Low | Low |
| Li J.*et al.* (2025) | Moderate | Low | Low | Low | Low | Low | Low |
| Chen *et al.* (2025) | Moderate | Low | Low | Low | Low | Low | Low |

**Table S4 Quality assessment of the included RCT study**

| Studies | Bias arising from the randomization process | Bias due to deviations from intended interventions | Bias due to missing outcome data | Bias in measurement of the outcome | Bias in selection of the reported result |
| --- | --- | --- | --- | --- | --- |
| Wei *et al.* (2025) | Some concerns | Low | Low | Low | Low |

**Table S5 Results of Egger’s test**

| outcomes/subgroup | *P*-value | Interpretation |
| --- | --- | --- |
| **ORR** | | |
| Observation | 0.7507 | No bias detected |
| Ablation | 0.0559 | No bias detected |
| Conization | 0.6871 | No bias detected |
| **CR 6mon** | | |
| Observation | 0.8710 | No bias detected |
| Ablation | Less than five studies | |
| Conization | 0.9707 | No bias detected |
| **CR 12mon** | | |
| Observation | 0.7507 | No bias detected |
| Ablation | Less than five studies | |
| Conization | Less than five studies | |
| **HPV clearance rate 6mon** | | |
| Observation | 0.0188 | potential publication bias |
| Ablation | 0.6404 | No bias detected |
| Conization | 0.8988 | No bias detected |
| **HPV clearance rate 12mon** | | |
| Observation | 0.4133 | No bias detected |
| Ablation | Less than five studies | |
| Conization | Less than five studies | |
| **Single-arm ORR** | | |
| LSIL | 0.1322 | No bias detected |
| HSIL | 0.2243 | No bias detected |
| **Single-arm CR 12mon** | | |
| LSIL | 0.0990 | No bias detected |
| HSIL | Less than five studies | |
| **Single-arm HPV clearance rate 12mon** | | |
| LSIL | 0.1507 | No bias detected |
| HISL | Less than five studies | |

**Table S6 Detail data for adverse event**

| Adverse event | Study | Intervention of control | PDT | Control |
| --- | --- | --- | --- | --- |
| Pain | Ma *et al.* (2021) | ablation | 21/87 | 21/97 |
|  | Wang. X. *et al.* (2024) | conization | 17/42 | 50/50 |
|  | Yang *et al.* (2025) | conization | 3/60 | 5/60 |
|  | Su *et al.* (2024) | ablation | 3/133 | 5/58 |
|  | Lu *et al.* (2025) | conization | 4/50 | 10/66 |
| Increased vaginal secretions | Ma *et al.* (2021) | ablation | 20/87 | 95/97 |
|  | Wang. L. *et al.* (2024) | conization | 20/74 | 37/116 |
|  | Yang *et al.* (2025) | conization | 4/60 | 5/60 |
|  | Su *et al.* (2024) | ablation | 8/133 | 7/58 |
|  | Lu *et al.* (2025) | conization | 42/50 | 60/66 |
| Pruritus | Ma *et al.* (2021) | ablation | 5/87 | 3/97 |
|  | Wang. L. *et al.* (2024) | conization | 7/74 | 4/116 |
|  | Su *et al.* (2024) | ablation | 4/133 | 3/58 |
| bleeding | Zhang *et al.* (2024) a | ablation | 0/86 | 4/110 |
|  | Zhang *et al.* (2024) b | conization | 0/86 | 2/89 |
|  | Ma *et al.* (2021) | ablation | 5/87 | 10/97 |
|  | Wang. L. *et al.* (2024) | conization | 0/74 | 12/116 |
|  | Wang. X. *et al.* (2024) | conization | 4/42 | 50/50 |
|  | Yang *et al.* (2025) | conization | 0/60 | 3/60 |
|  | Su *et al.* (2024) | ablation | 0/133 | 1/58 |
|  | Chen *et al.* (2022) | ablation | 1/62 | 2/53 |
|  | Lu *et al.* (2025) | conization | 0/50 | 20/66 |
| Burning | Wang. L. *et al.* (2024) | conization | 9/74 | 13/116 |
| Abdominal distension | Chen *et al.* (2024) | conization | 16/72 | 28/76 |
| Dysmenorrhea | Chen *et al.* (2022) | ablation | 1/62 | 1/53 |

**Table S7 GRADE assessment**

| outcomes | group | study design | Risk of Bias | Inconsistency | Imprecision | Indirectness | Publication Bias | Quality |
| --- | --- | --- | --- | --- | --- | --- | --- | --- |
| ORR | observation | NRCT  +RCT | not serious | very serious | serious | not serious | not serious | Very low |
|  | ablation | NRCT | not serious | not serious | serious | not serious | not serious | Low |
|  | conization | NRCT | not serious | serious | very serious | not serious | not serious | Very low |
| CR-6mon | observation | NRCT  +RCT | not serious | serious | serious | not serious | not serious | Low |
|  | ablation | NRCT | not serious | not serious | very serious | not serious | not serious | Very low |
|  | conization | NRCT | not serious | serious | very serious | not serious | not serious | Very low |
| HPV-6mon | observation | NRCT  +RCT | not serious | very serious | serious | not serious | serious | Very low |
|  | ablation | NRCT | not serious | not serious | serious | not serious | not serious | Very low |
|  | conization | NRCT | not serious | not serious | very serious | not serious | not serious | Very low |
| recurrence | observation | NRCT | not serious | not serious | very serious | not serious | not serious | Very low |
|  | ablation | NRCT | not serious | not serious | not serious | not serious | not serious | Moderate |
|  | conization | NRCT | not serious | not serious | very serious | not serious | not serious | Very low |
| progression | observation | NRCT | not serious | not serious | not serious | not serious | not serious | Moderate |
|  | ablation | NRCT | not serious | not serious | very serious | not serious | not serious | Very low |

**Table S8 Assessment of potential heterogeneity sources**

| Studies | Potential source of heterogeneity | | | | | |
| --- | --- | --- | --- | --- | --- | --- |
|  | Age, mean±SD  （PDT/control） | lesion location/extent | Treatment sessions | HPV genotype | PDT protocols | Follow-up duration |
| Chen et al. (2023) | 31.30±7.12/ 31.68±7.16 | TZ1and2 | 3 | HPV16/18 and non-HPV16/18 | 20%ALA  100J/m^2^ 635nm | 12mon |
| Qi et al. (2024) | 43.3±11.65/ 42.7±11.97 | Vaginal wall and endocervical lesions included | 3-6 | HPV16/18 and non-HPV16/18 | 20%ALA  100J/m^2^ 635nm | 12mon |
| Chen et al. (2024) | 34.57±5.66/ 34.57±4.98 | Not reported | 3 | HPV16/18 and non-HPV16/18 | 20%ALA  100J/m^2^ 635nm | 12mon |
| Jiang et al. (2023) | 35.45±10.25/ 34.33±7.0 | Not reported | 3-6 | HPV16/18 and non-HPV16/18 | 20%ALA  80J/m^2^ 633nm | 12mon |
| Wei et al. (2025) | 39.46±7.61/ 39.22±6.68 | Exclude vulvar lesions | 6 | HPV16/18 and non-HPV16/18 | 20%ALA  100J/m^2^ 635nm | 12mon |
| Chen et al. (2025) | 34/38 | TZ3, Exclude vulvar lesions | 6 | HPV16/18 and non-HPV16/18 | 20%ALA  100J/m^2^ 635nm | 12mon |

**Fig. S1 sensitivity analysis**


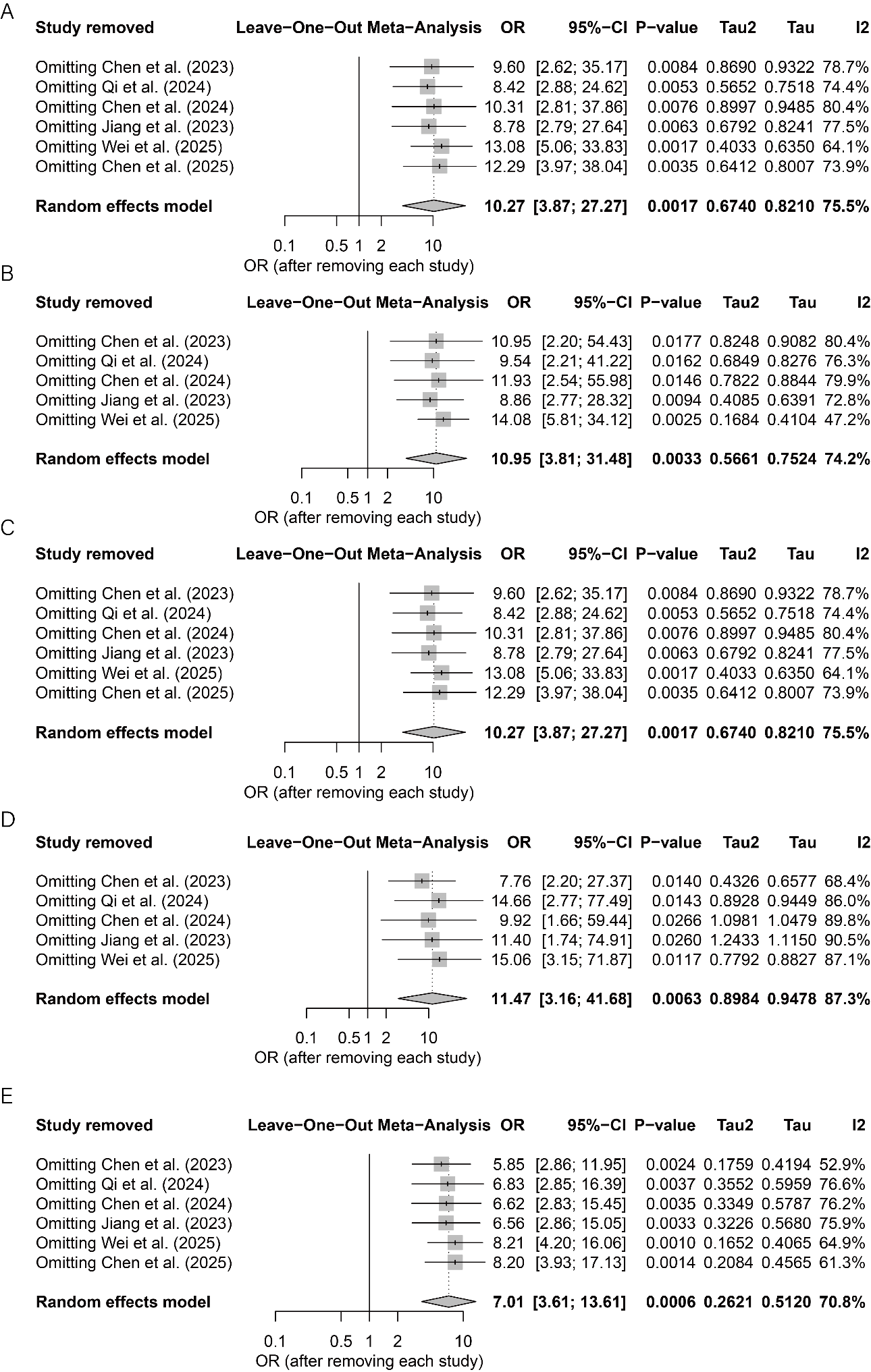


A. Sensitivity analysis for observation group in ORR B. Sensitivity analysis for observation group in 6-month follow-up CR rate C. Sensitivity analysis for observation group in 12-month follow-up CR rate D. Sensitivity analysis for observation group in 6-month follow-up HPV clearance rate E. Sensitivity analysis for observation group in 12-month follow-up HPV clearance rate

**Fig. S2 Forest plot of ORR stratified by treatment sessions in the observation subgroup**


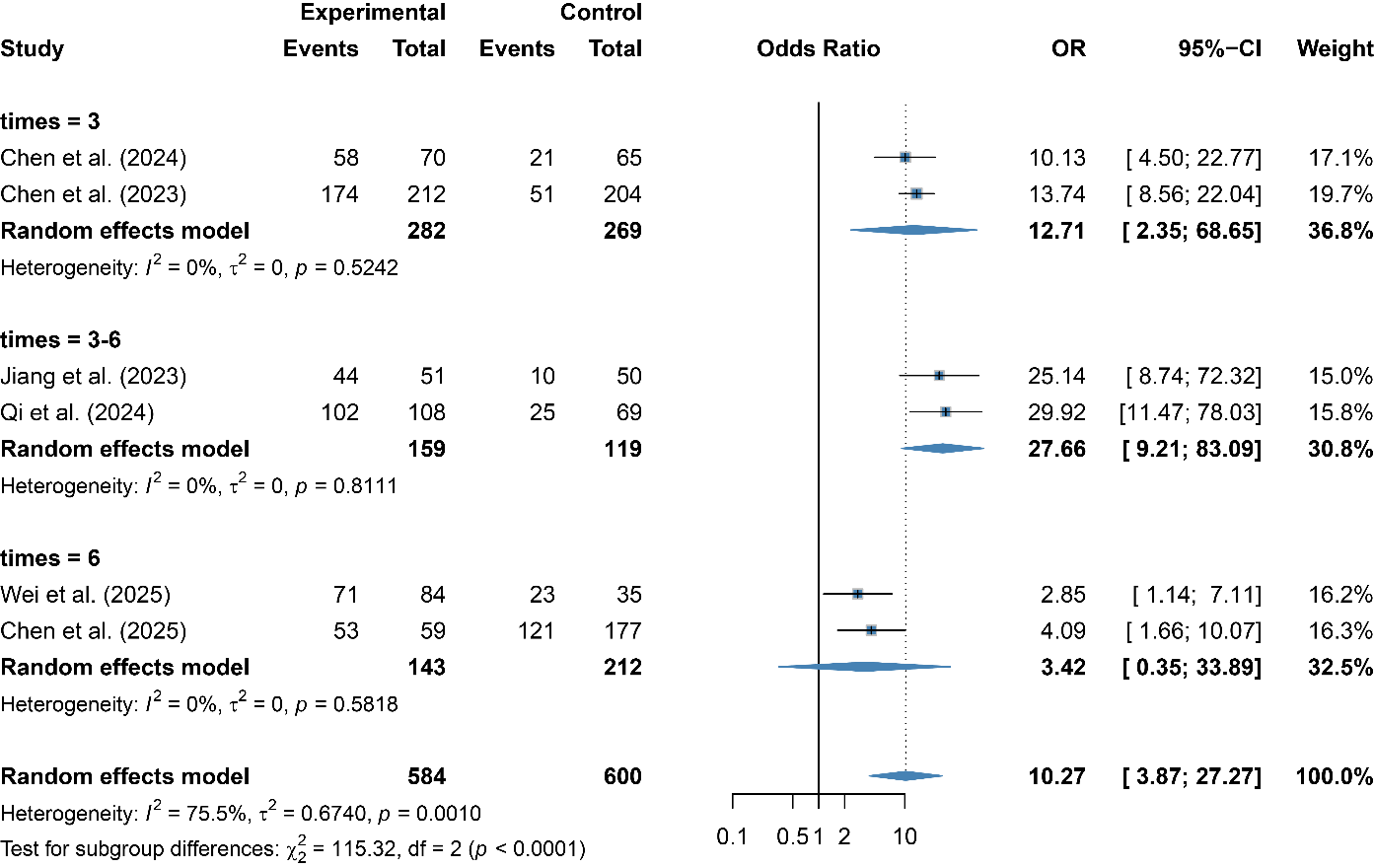


**Fig. S3 Forest plot comparing the ORR between 5-ALA PDT and control treatments stratified by lesion grade: (A) LSIL; (B) HSIL**


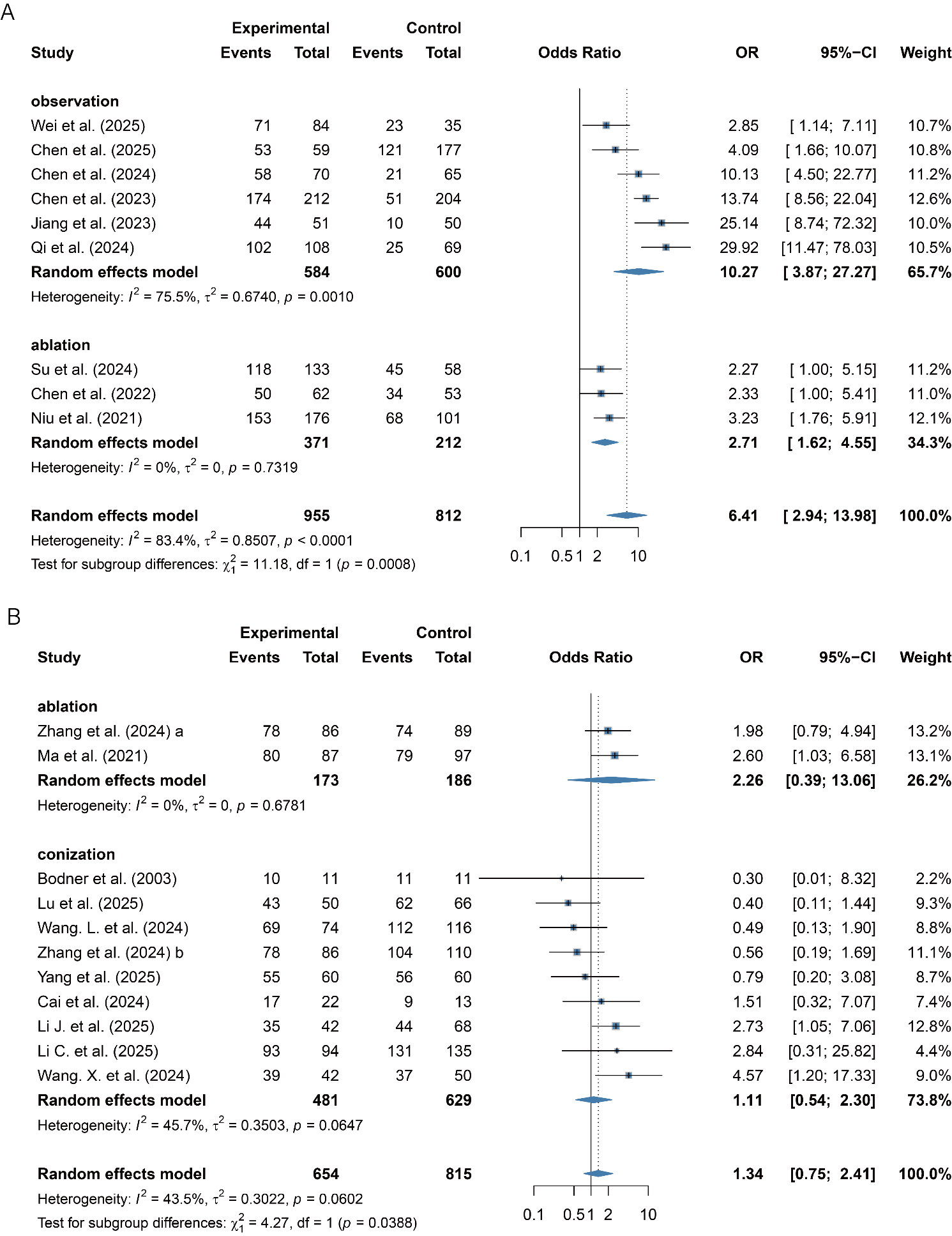


**Fig. S4 funnel plot for ORR**


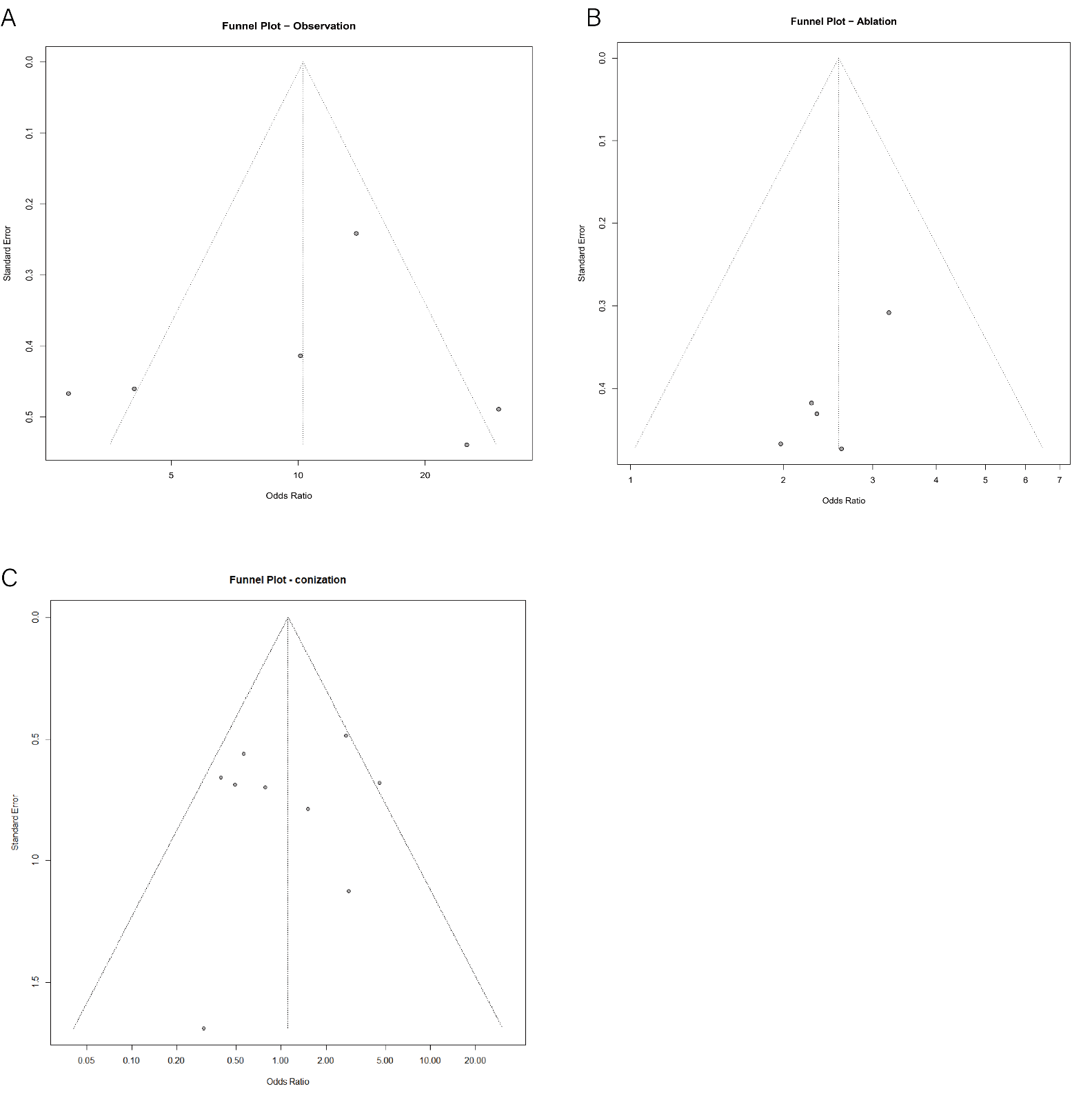


A. Funnel plot for observation group B. funnel plot for ablation group C. funnel plot for conization group

**Fig. S5 Forest plot comparing the 6-month CR rate between 5-ALA PDT and control treatments stratified by lesion grade: (A) LSIL; (B) HSIL**


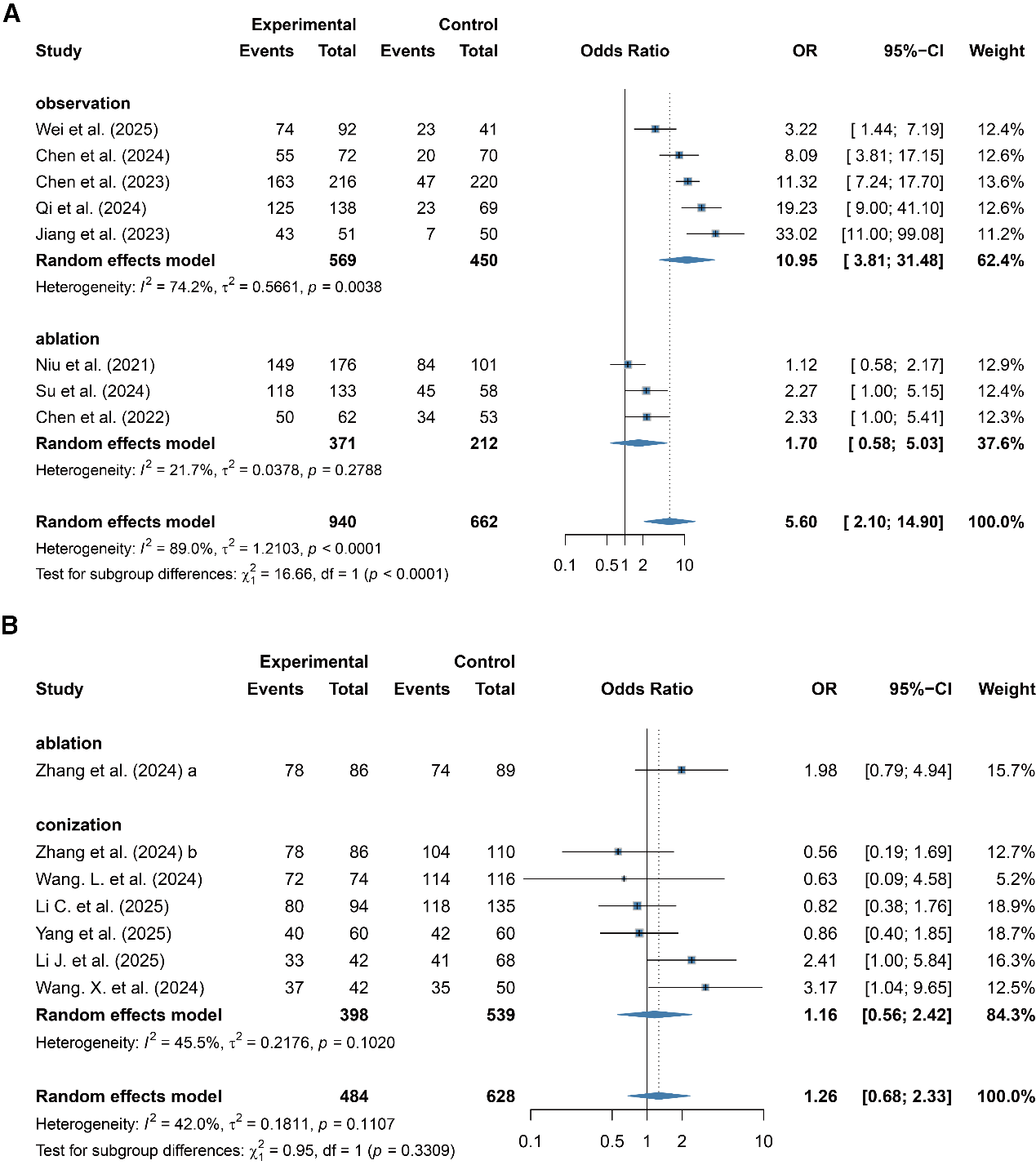


**Fig. S6 Forest plot comparing the 6-month HPV clearance rate between 5-ALA PDT and control treatments stratified by lesion grade: (A) LSIL; (B) HSIL**


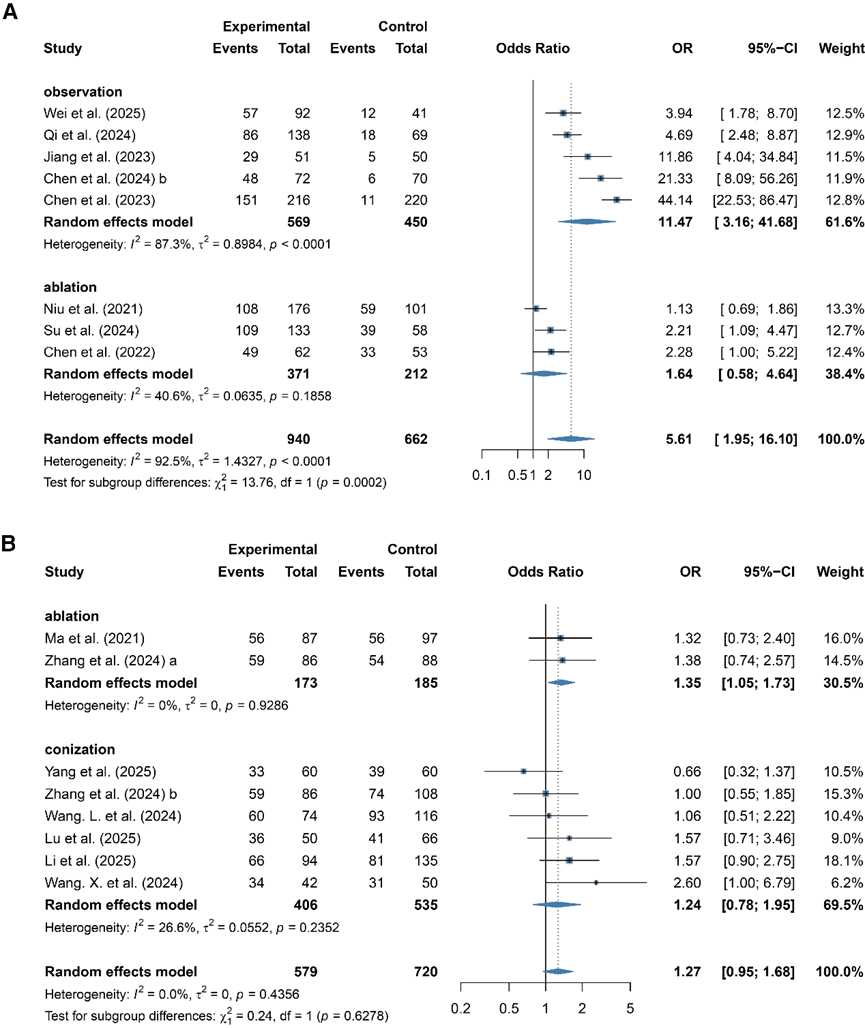


**Fig. S7 Forest plot of 12-month HPV clearance stratified by treatment sessions in the observation subgroup**


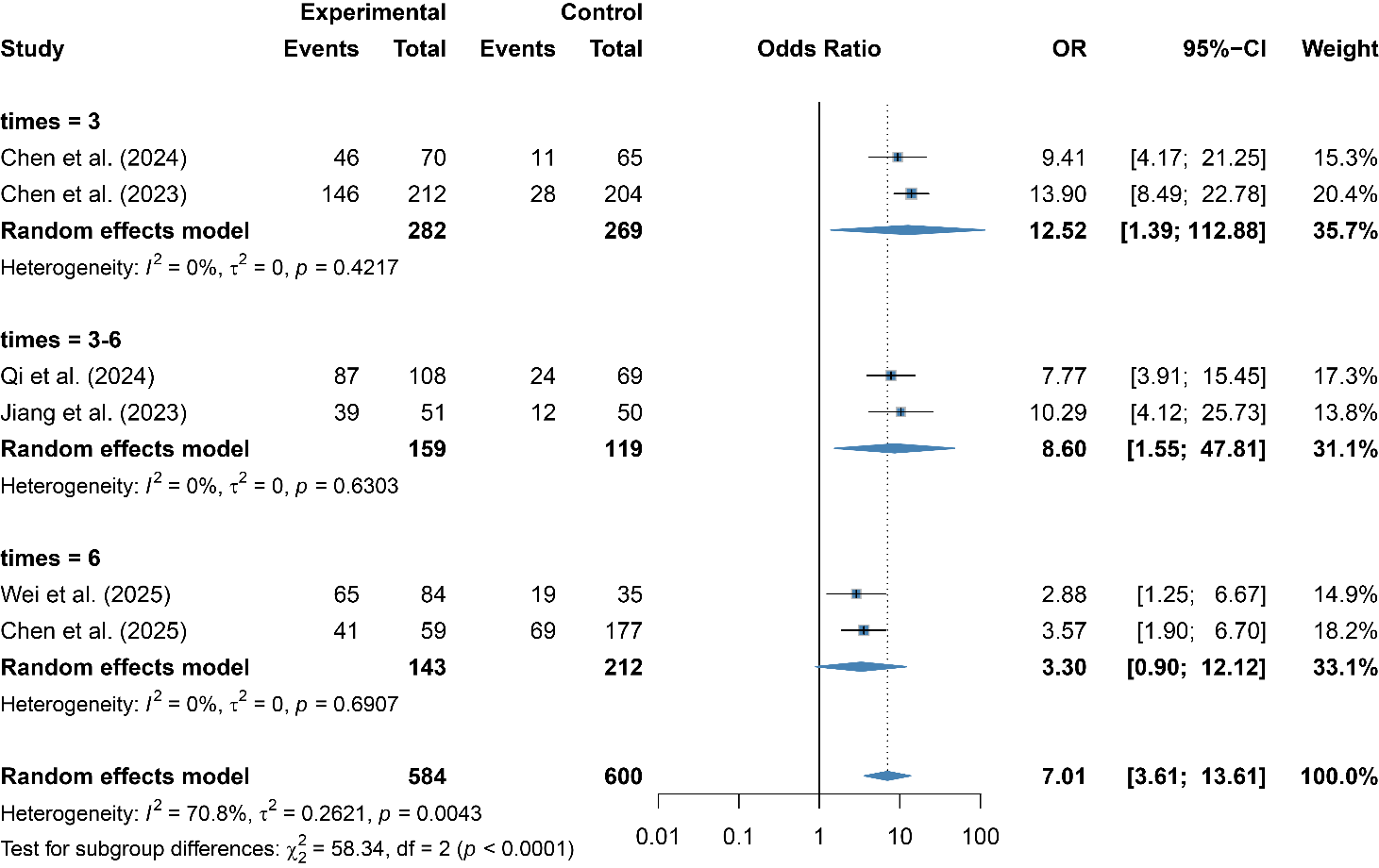


**Fig. S8 Forest plot for HPV16/18 clearance rate between 5-ALA PDT and control treatments**


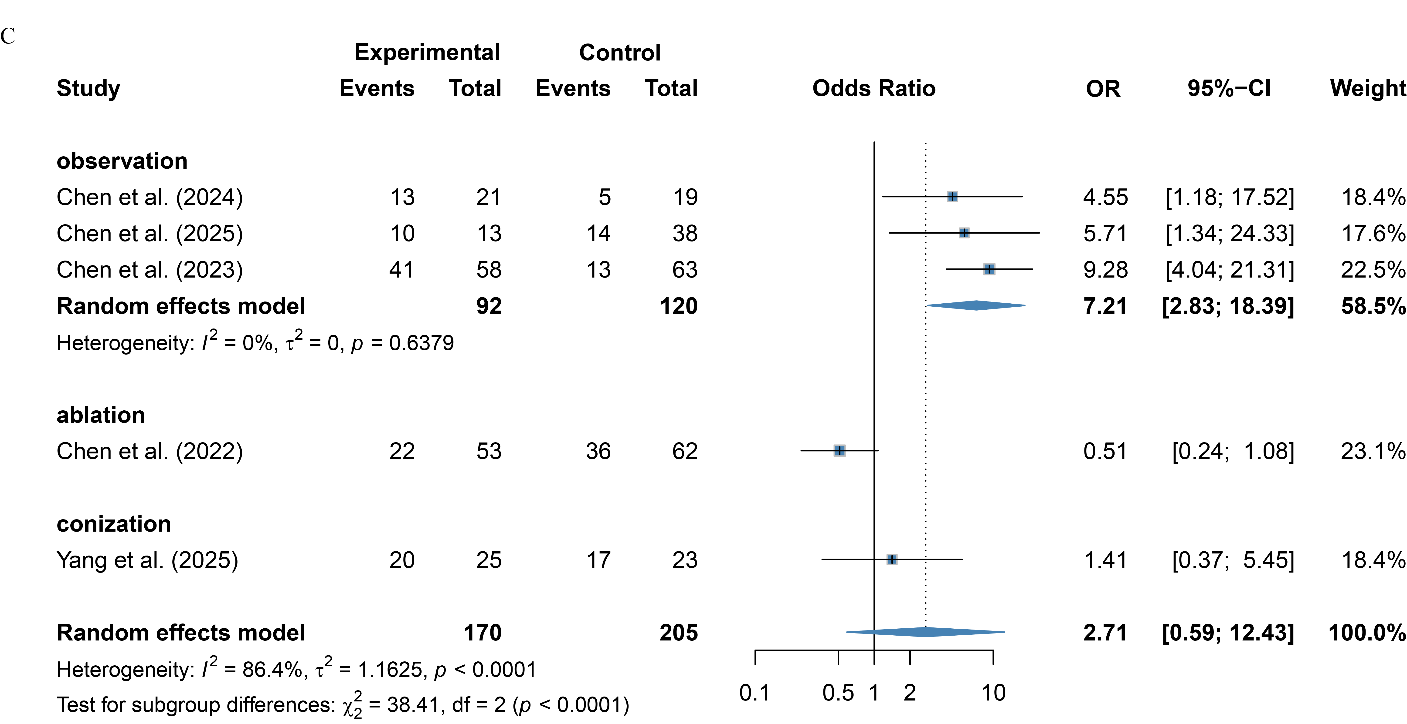


**Fig. S9 Forest plot comparing the ORR stratified by lesion extent between 5-ALA PDT and control treatments**


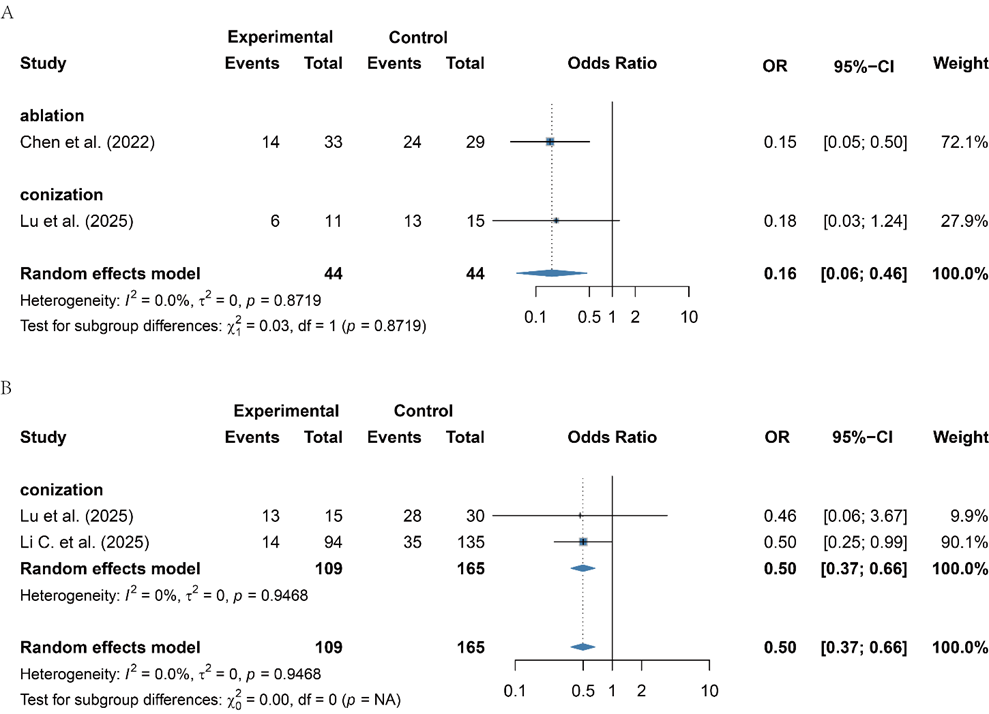


A. Forest plot for ORR in patients with cervical canal lesion B. Forest plot for ORR in patients with gland lesion

**Fig. S10 Forest plot comparing the recurrence rate between 5-ALA PDT and control treatments**

**
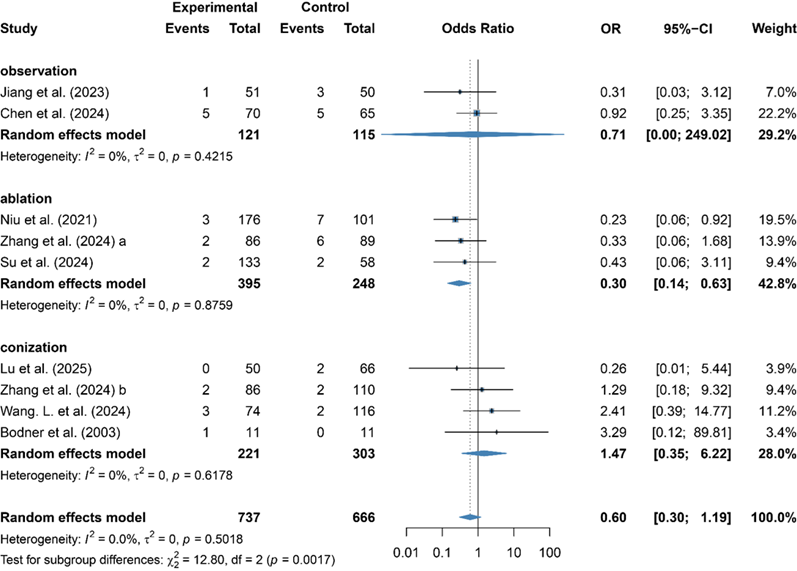
**

**Fig. S11 Forest plot comparing the progression rate between 5-ALA PDT and control treatments**

**
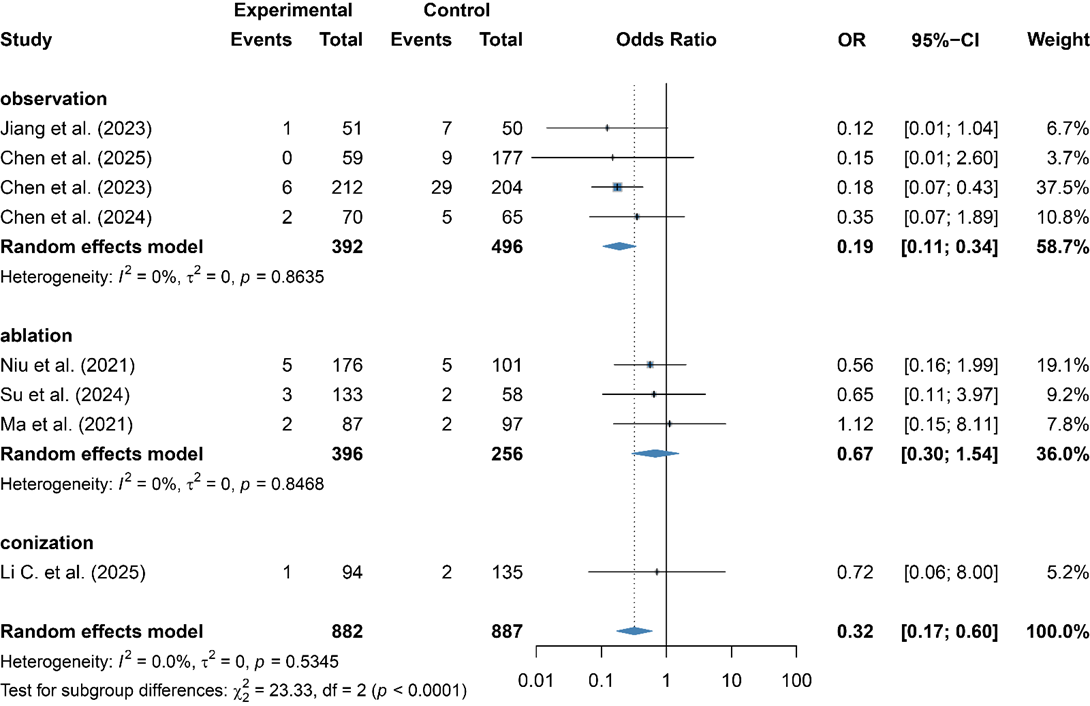
**

**Fig. S12 Forest plot of ORR following 5-ALA PDT according to the number of treatment sessions**


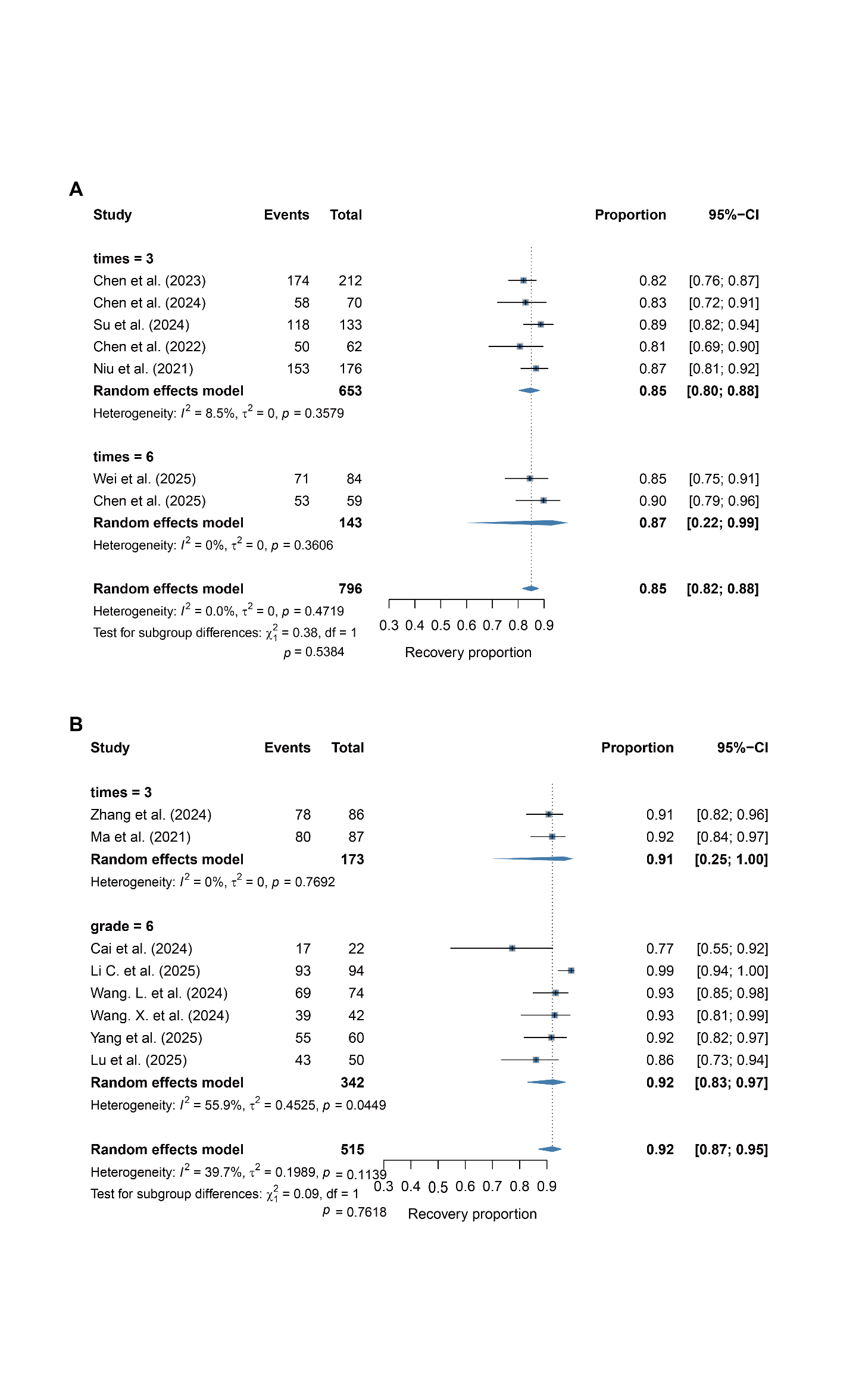


（A）ORR for LSIL（B）ORR for HSIL

**Fig. S13 Forest plot of 12-month CR rate after 5-ALA PDT in patients with different lesion grades**


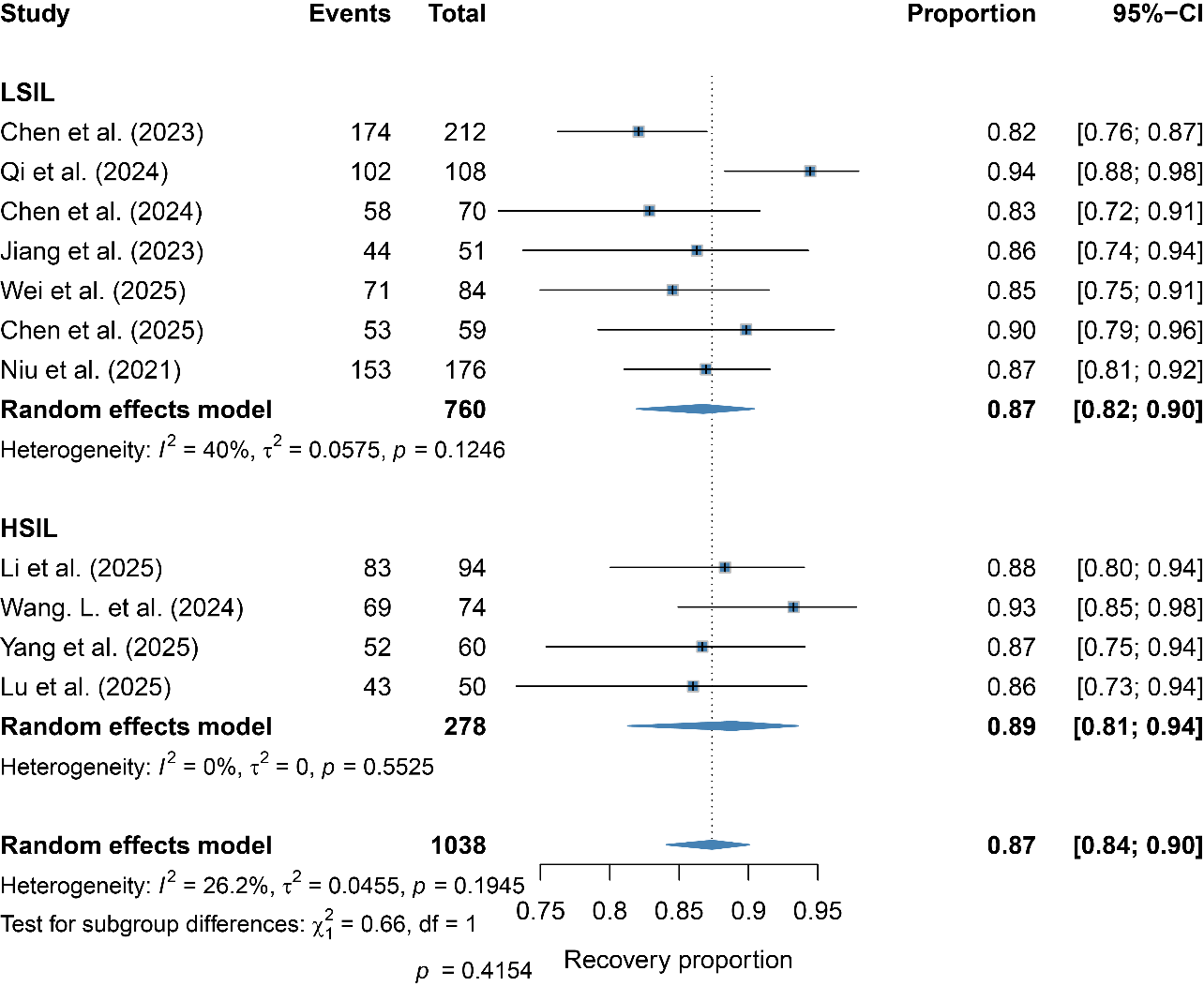


**Fig. S14 Forest plot of 12-month HPV clearance rate after 5-ALA PDT in patients with different lesion grades**

**
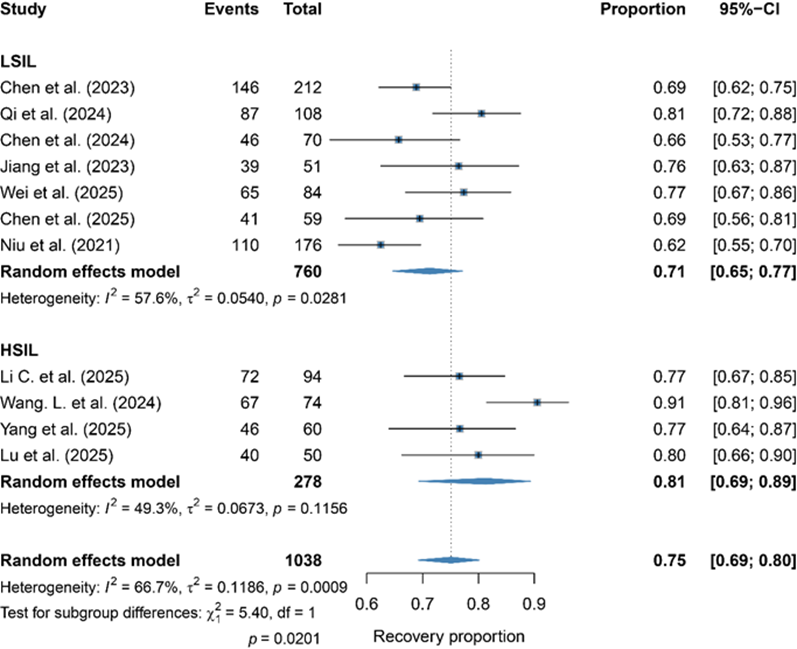
**
